# Macrophages promote collagen deposition through circadian regulation of fibroblasts

**DOI:** 10.1016/j.isci.2026.117573

**Published:** 2026-09-21

**Authors:** Katherine Lowles, Xin-Yang Zhang, Xue-Feng Yao, Marie F.A. Cutiongco, Joshua J. Hughes, Shi-Yang Li, John Knox, Madeleine Coy, Wei-Hsiang Lin, Andrew S. MacDonald, Karl E. Kadler, Qing-Jun Meng, Tracy Hussell, Joan Chang

**Affiliations:** 1 Manchester Cell-Matrix Centre, Division of Molecular and Cellular Function, Faculty of Biology, Medicine and Health, University of Manchester, Manchester, UK; 2 Division of Cell Matrix Biology and Regenerative Medicine, Faculty of Biology, Medicine and Health, University of Manchester, Manchester, UK; 3 Division of Infection, Immunity and Respiratory Medicine, Faculty of Biology, Medicine and Health, University of Manchester, Manchester, UK; 4 Genome Editing Unit, Faculty of Biology, Medicine and Health, University of Manchester, Manchester, UK

**Keywords:** collagen, extracellular matrix, circadian rhythm, macrophages

## Abstract

Fibroblast collagen deposition is regulated by macrophages and the circadian rhythm; understanding how these processes interact provides insights to collagenous tissue homeostasis. Here, we reveal that macrophage-fibroblast interaction enhances collagen deposition and requires a functional circadian rhythm. Macrophage polarization status determines how fibroblast responses were elicited, where naive and pro-inflammatory macrophages require direct cell-cell contact, and anti-inflammatory macrophages secrete soluble factors. One key secreted factor identified by mass spectrometry proteomics analysis is PDGFA. Crucially, macrophages lacking PDGFA, or treatment with PDGFRa inhibitor, showed diminished ability to modulate fibroblast circadian rhythms and collagen production. Impaired circadian rhythms in either cell type also led to a reduced collagen fibrillogenesis response. These results confirmed that the collagen deposition pathway is under circadian clock control in lung fibroblasts, and demonstrated that macrophages can re-synchronize fibroblast circadian rhythms to promote collagen deposition and extracellular remodeling, which are implicated in processes such as wound healing responses.

## Highlights

• Macrophages induce fibroblast circadian rhythms, driving collagen fibrillogenesis
• PDGFA/PDGFRa signaling axis is central to macrophage-fibroblast crosstalk
• Loss of circadian rhythms in either cell types abrogate collagen fibrillogenesis
• This macrophage-fibroblast crosstalk may be important in normal wound healing

## Graphical abstract

**Figure:**
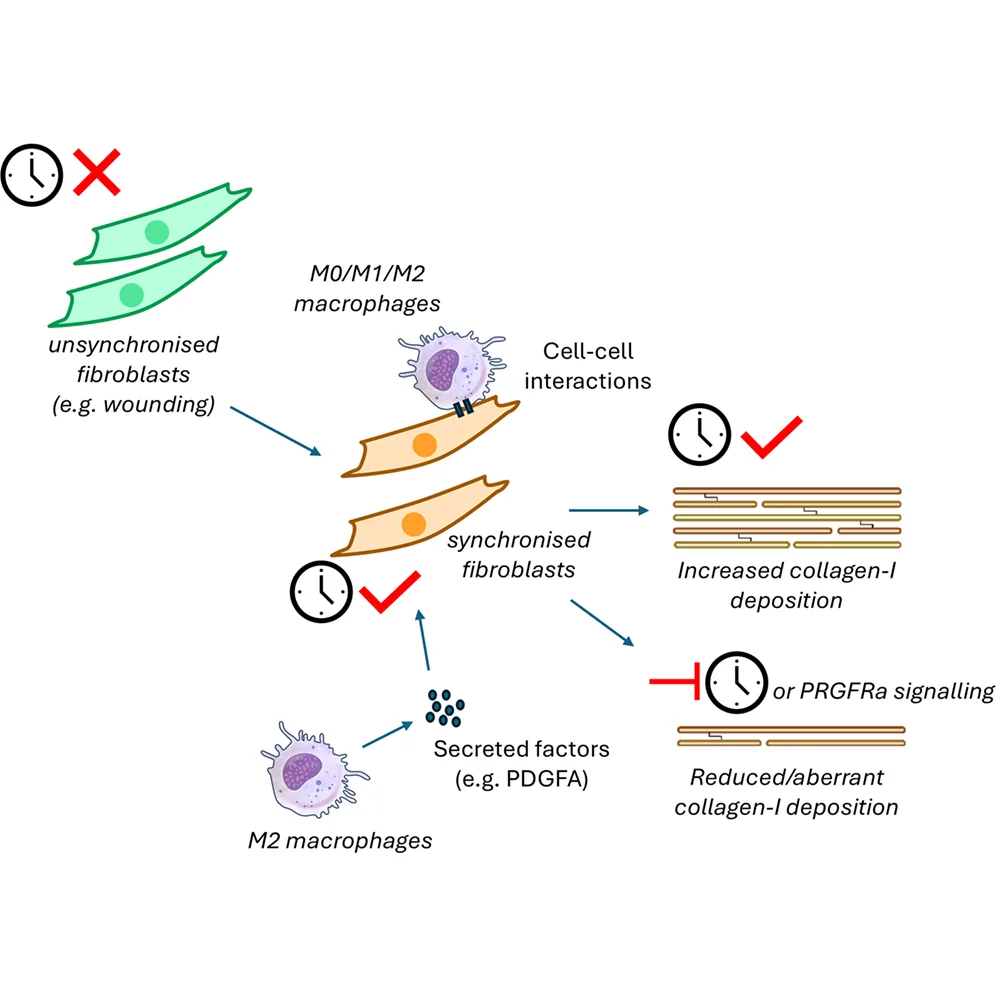

## Teaser

Immunology; Natural sciences; Biological sciences; Immunology; Cell biology

## Introduction

Type-I collagen (collagen) is one of the most abundant extracellular matrix (ECM) proteins, and its homeostatic control is crucial for tissue health. Dysregulation of collagen leads to conditions such as fibrosis and chronic wounds.^1^ The key cells involved in collagen regulation are fibroblasts and macrophages. Fibroblasts are the predominant collagen-producing cells, while macrophages play a regulatory role by secreting cytokines and growth factors that influence collagen synthesis in fibroblasts.^2^^,^^3^ Intriguingly, macrophages have also been reported to directly deposit collagen at wound sites, suggesting additional regulatory roles in the ECM. However, it is unclear if this deposition is also observed under homeostatic conditions.^4^

Recent studies have demonstrated that in collagen-rich tissues, such as tendons, there is a daily surge of collagen production. Certain genes involved in collagen biosynthesis—particularly those that regulate trafficking through the secretory pathway—are controlled by the circadian clock.^5^ Around 50% of mammalian genes are rhythmically expressed with any 24-h cycle, thus maintaining appropriate rhythms in these clock-controlled genes (CCGs) is critical for homeostasis and health.^6^^,^^7^ Uncoupling CCGs from the circadian clock results in impaired wound healing and contributes to a host of ECM-related diseases, including lung fibrosis.^8^^,^^9^ It is crucial to understand the mechanisms that regulate rhythmic collagen secretion in order to determine how these processes are disrupted under non-homeostatic conditions such as during injury responses, thus guiding the development of therapeutic strategies to restore a healthy, balanced state.

Macrophages play a pivotal role in tissue remodeling and repair by influencing fibroblast activity and collagen deposition.^10^ Through direct cell-cell interactions and the secretion of soluble factors, macrophages modulate fibroblast behavior, including their capacity to produce and organize collagen within the ECM.^11^ This crosstalk is essential for maintaining tissue homeostasis and responding to injury, and disruption may drive fibrotic responses. Importantly, macrophage polarization states—such as the pro-inflammatory M1 and pro-reparative M2 phenotypes—differentially regulate fibroblast activity, suggesting that macrophages fine-tune collagen dynamics depending on the physiological or pathological context.^12^^,^^13^ As macrophages are known to localize to sites of high ECM/collagen deposition and remodeling, this raises the possibility that macrophages may also interfere with the fibroblast circadian clock to synchronize and enhance rhythmic collagen production.

We hypothesized that macrophages influence collagen deposition in fibroblasts by modulating the fibroblast circadian clock. To test this, we assessed the impact of macrophages on collagen deposition using immunofluorescence (IF) imaging and investigated circadian rhythms in fibroblasts using the *Per2*-Luc circadian reporter (where luciferase is used as a readout for the circadian core clock protein Per2^14^). Here, we showed for the first time that macrophages, regardless of polarization status (M0—naive; M1— lipopolysaccharide (LPS) and interferon-γ (IFN-γ) stimulated; M2—IL-4 and IL-13 stimulated), enhanced circadian rhythms of lung fibroblasts. Furthermore, we observed an increased collagen deposition in co-cultures. Intriguingly, M0 and M1 macrophages require cell-cell contact with fibroblasts to exert their influence, whereas secreted factors from M2 macrophages alone are sufficient to drive collagen fibrillogenesis and circadian rhythm induction. Using mass spectrometry (MS) proteomics analysis, we identified platelet-derived growth factor subunit A (PDGFA) as a key M2 macrophage-secreted factor influencing fibroblast function. Notably, PDGFA alone can induce a circadian rhythm but is insufficient to promote collagen fibrillogenesis. Importantly, knocking out PDGFA in macrophages abrogated their ability to promote collagen deposition and enhance circadian rhythms in fibroblasts; this effect was also seen when treating fibroblasts-macrophage co-culture with the specific platelet-derived growth factor receptor alpha (PDGFRa) inhibitor, avatinib, highlighting the importance of macrophage-induced PDGFA signaling in fibroblast circadian rhythms and collagen functions. Additionally, the macrophage-induced effects were dependent on a functional circadian rhythm in both fibroblasts and macrophages; interestingly, macrophages can shift the phase of the fibroblasts regardless of the circadian phase or synchronicity of either cell type. Together, these results demonstrate that macrophages promote collagen deposition in fibroblasts by inducing circadian rhythms, with M2-derived PDGFA playing a key role in this process.

## Results

### Macrophages induce robust circadian rhythms in fibroblasts via myeloid-specific mechanisms

The interaction between macrophages and the circadian clock in fibroblasts was explored using fibroblasts isolated from the *Per2*-Luc (clock protein Period2 fused with luciferase protein) mice, where luminescence is released in the presence of luciferin and represents a direct readout of the abundance of Per2 protein; this provides real-time tracking of the circadian rhythm in fibroblasts.^15^ Human cell line THP-1 monocytes and macrophages, as well as primary alveolar macrophages (AMs) were added to *Per2*-Luc fibroblasts approximately 24 h after bioluminescence recording began.

Untreated *Per2*-Luc lung fibroblasts (black trace, Figure 1A) showed an initial small peak that represented the overall sum of endogenous circadian rhythms of lung fibroblasts in culture, which are synchronized at low levels, likely through mechanical force exerted during media change and placement of culture dishes in the LumiCycle apparatus. This synchronization rapidly diminished, reflecting desynchronization caused by weak intercellular clock-clock coupling and cell division.^16^

**Figure 1:**
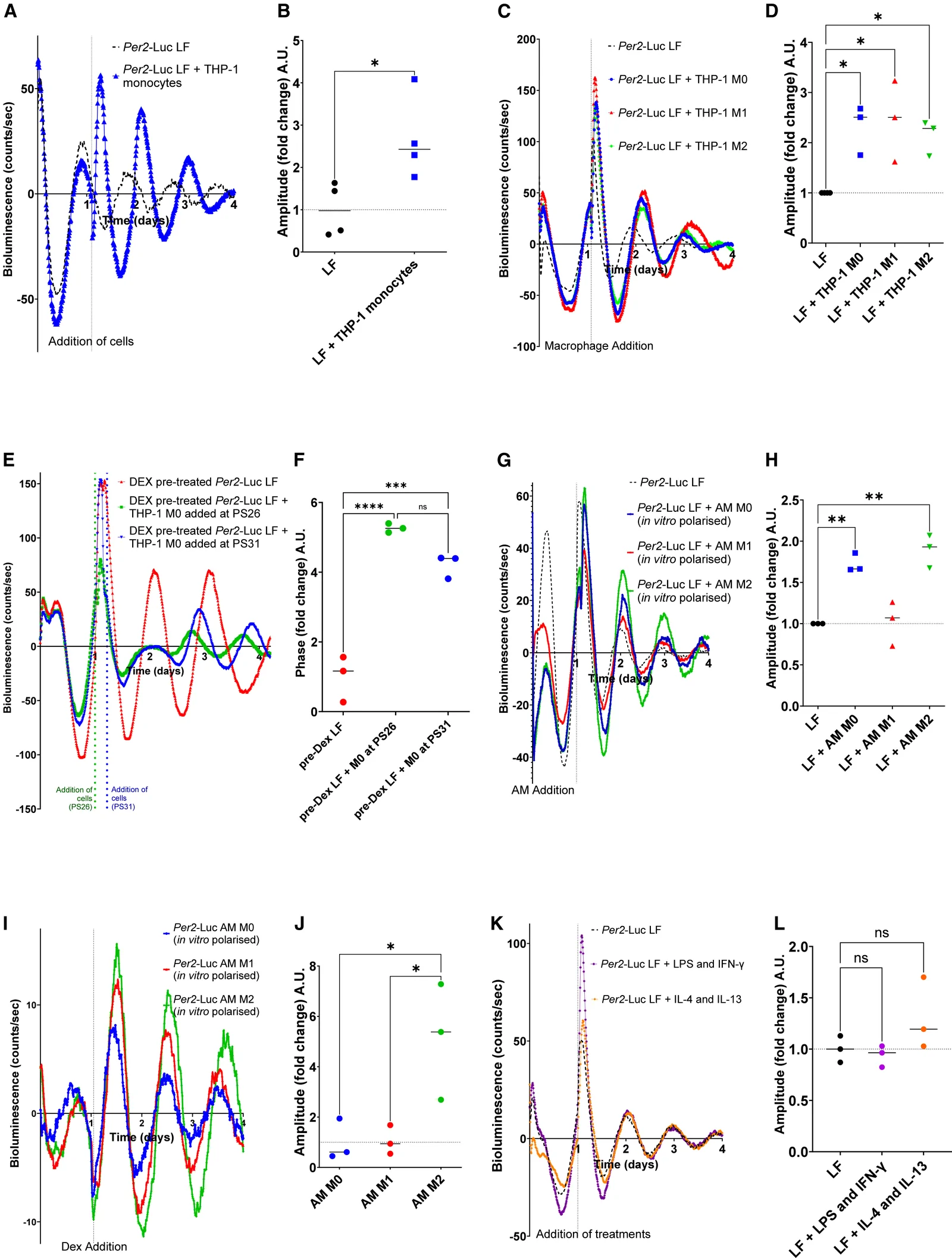
Bioluminescence readings of *Per2*-Luc lung fibroblasts with polarized THP-1 macrophages (A) Addition of THP-1 monocytes induced a robust circadian rhythm in fibroblasts. Representative of *n* = 4. (B) Amplitude fold change was shown to be significant following monocyte addition. Data are represented as mean ± SEM. *n* = 4 biological repeats, ∗*p* < 0.05, unpaired two-tailed *t* test. (C) Addition of M0, M1, and M2 polarized THP-1 macrophages induced robust rhythms in *Per2*-Luc fibroblasts, significantly increasing bioluminescence amplitude. Representative of *n* = 3. (D) Amplitude fold change showed significant increases with M0, M1, and M2, compared to LF alone. Data are represented as mean ± SEM. *n* = 3 biological repeats, ∗*p* < 0.05, one-way ANOVA. (E) Addition of M0 THP-1-derived macrophages at different phases (26 h post-synchronization, PS26; 31 h post-synchronization, PS31) representing the ascending and descending phase of the circadian oscillation. Representative of *n* = 3. (F) Period measurement showed that addition of macrophages at PS26 and PS31 both showed significantly altered phases. Data are represented as mean ± SEM. *n* = 3 biological repeats, ∗∗∗∗*p* < 0.001, ∗∗∗*p* < 0.005, one-way ANOVA. (G) Addition of *in vitro* polarized primary murine AMs (M0 and M2) also induced robust rhythms in fibroblasts, with M2 having the greatest effect. Representative of *n* = 3. (H) Amplitude fold change showed significant increases with M0 and M2. Data are represented as mean ± SEM. *n* = 3 biological repeats, ∗∗*p* < 0.01, one-way ANOVA. (I) M2-polarized *Per2*-Luc macrophages sustained the circadian rhythm with the highest amplitude following dexamethasone addition. Representative of *n* = 3. (J) Amplitude fold change analysis revealed that M2 macrophages significantly enhanced circadian amplitude compared to M0 and M1 macrophages. Data are represented as mean ± SEM. *n* = 4 biological repeats, ∗*p* < 0.05, one-way ANOVA. (K) M1-and M2-polarizing factors were added to lung. Neither LPS and IFN-ɣ, nor IL-4 and IL-13 (M1 and M2 polarizing factors, respectively) increased circadian amplitude. Representative of *n* = 3. (L) Quantification of amplitude fold change showed there was no significant increase in fibroblast circadian amplitude following addition of M1-and M2-polarizing factors. Data are represented as mean ± SEM. *n* = 3 biological repeats, one-way ANOVA.

Addition of THP-1 monocytes (blue trace, Figure 1A) after ∼24 h resulted in a sustained and robust circadian rhythm in fibroblasts for over 2 days. Quantification of amplitude changes (Figure 1B) demonstrated that macrophages significantly enhanced circadian rhythm amplitude. In contrast, the addition of the epithelial Eph4 cells did not significantly increase amplitude (Figures S1A and S1B), suggesting a macrophage-specific effect.

To confirm if THP-1 macrophages can also induce a rhythm in fibroblasts, we differentiated them first to M0 using phorbol 12-myristate 13-acetate (PMA), followed by polarization to M1 (pro-inflammatory, defined as 48-h treatment with LPS and IFN-γ in this study), or M2 (anti-inflammatory, defined as 48-h treatment with IL-4 and IL-13 in this study) states. This allowed us to determine whether effects on fibroblast circadian rhythms are dependent on macrophage polarization status.

Polarization was validated by qPCR analysis of classical M1 (CD64) and M2 (TGF-β) gene markers (Figures S1C and S1D). M0, M1, and M2 macrophages all significantly increased circadian rhythm amplitude in fibroblasts (Figures 1C and 1D), with no significant differences between polarization states.

We then asked if the circadian status of the macrophages influence their synchronization ability. Macrophages were synchronized with dexamethasone, a glucocorticoid known to synchronize circadian rhythm, for 1 h or 24 h prior to addition to *Per2*-Luc fibroblasts.^17^ The synchronization status of macrophages did not alter their rhythm-enhancing effects on fibroblasts; unsynchronized, 1-h synchronized, and 24-h synchronized macrophages all induced significant amplitude increases (Figures S1E and S1F). Further, macrophages added at different phases of the fibroblast circadian cycle caused a significant phase shift (Figures 1E and 1F), with no significant change in the period (Figure S1G); this suggests that it is the presence rather than timing of macrophages that drives a change in fibroblast circadian rhythm.

To confirm that induction of circadian rhythm in lung fibroblasts was not due to cross-species reactivity, we used primary murine AMs isolated from lungs of wild-type (WT) mice (i.e., no luciferase activity). Macrophages were then polarized *in vitro,* as confirmed by qPCR analyses (Figures S1H and S1I), and then added to *Per2*-Luc lung fibroblasts. Here, M2-polarized AMs induced the most robust rhythm in fibroblasts, followed by M0, while M1 AMs had minimal effect (Figures 1G and 1H). Similarly, *in vivo* polarized primary murine AMs were also assessed for their circadian-inducing effects. While M0, M1 and M2 macrophages all increased circadian amplitude in fibroblasts, only M2 *in vivo* polarized macrophages had a significant effect (Figures S1J and S1K).

To evaluate macrophage-autonomous rhythms, AMs from *Per2*-Luc transgenic mice were polarized and synchronized with dexamethasone. All AMs showed a synchronization response in their circadian rhythm; although amplitude measurements showed that, compared with M0 and M1, the amplitude increase in M2 AMs was significant. This suggests that M2 AMs inherently have the highest circadian amplitude and can better sustain a rhythm among the polarization statuses (Figures 1I and 1J). Importantly, addition of M1-polarizing factors (IFN-γ and LPS) or M2-polarizing factors (IL-4 and IL-13) to fibroblasts failed to enhance circadian amplitude, indicating it is a myeloid cell effect (Figures 1K and 1L).

Furthermore, THP-1 cells were transduced with a PER2-Luc2 plasmid in order to measure the circadian rhythms of M0 macrophages following the addition of WT fibroblasts (Figure S1L). Fibroblasts did not increase the circadian amplitude in M0 macrophages (Figure S1M).

We then asked in what physiological conditions a fibroblast might lose its synchronicity. It was previously reported that the speed of wound healing is dependent on circadian rhythm;^8^ here, we hypothesized that the act of wounding itself disrupt the circadian rhythm of fibroblasts. Confluent, Dex-synchronized fibroblasts were subjected to bioluminescence imaging followed by a scratch wound. Measurement of bioluminescence signal between control and scratched wells indicated a dampening of rhythm post-wounding, suggesting that wounding leads to disruption of fibroblast circadian rhythms which may then be reactivated by macrophage recruitment (Figure S1N).

Together, these results suggest that macrophages can induce a circadian rhythm in fibroblasts, and that this circadian effect is not reciprocal.

### Macrophage-secreted factors are responsible for the circadian-induction effects on fibroblasts

To investigate if macrophages influence circadian amplitude through the secretion of soluble factors, serum-free conditioned media (CM) from polarized macrophages was collected and added to *Per2*-Luc fibroblasts. Both THP-1 M0 and M2 CM increased circadian amplitude in the fibroblasts (Figure 2A). However, only CM from THP-1 M2 macrophages resulted in a statistically significant amplitude enhancement (Figure 2B).

**Figure 2:**
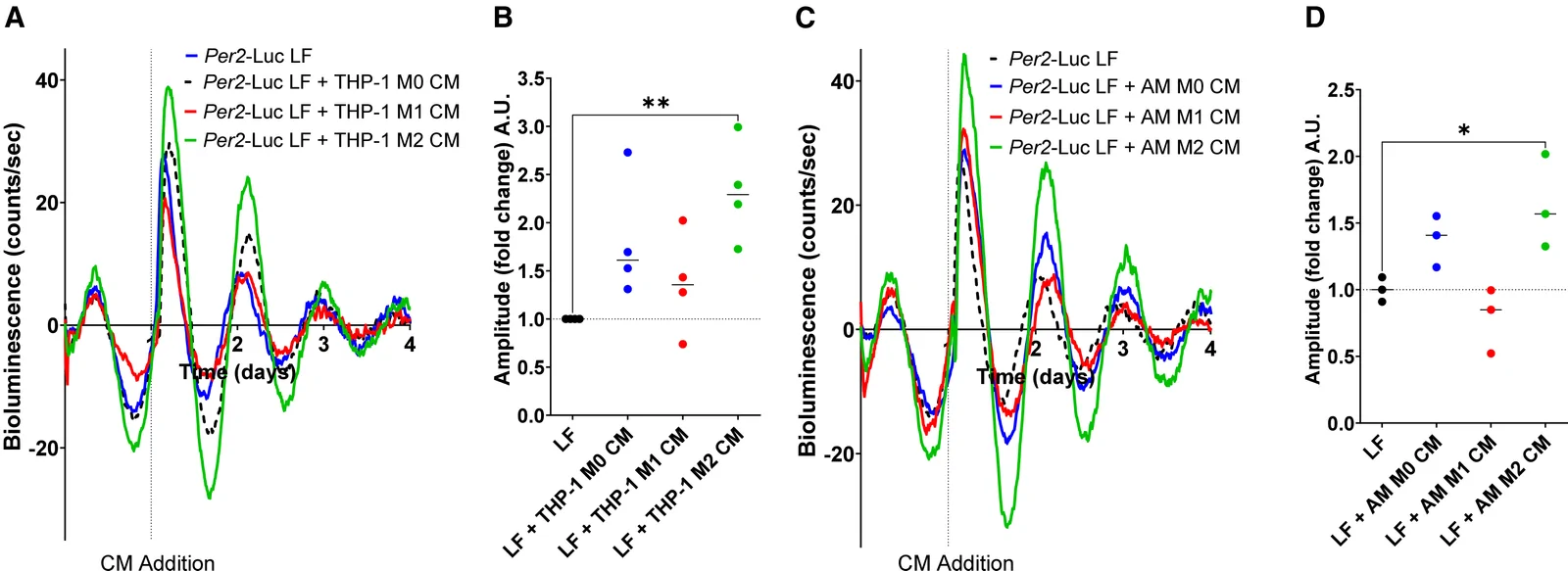
Bioluminescence readings of *Per2*-Luc lung fibroblasts with polarized THP-1 macrophage conditioned media (A) CM from polarized THP-1 macrophages was added to *Per2*-Luc lung fibroblasts. M2 macrophage CM induced a robust circadian rhythm in fibroblasts. Representative of *n* = 4. (B) Amplitude fold change analysis showed that while M0 CM and M2 CM increased circadian amplitude, only the increase with M2 CM was statistically significant compared to fibroblasts without CM. Data are represented as mean ± SEM. *n* = 4 biological repeats, ∗∗*p* < 0.01, one-way ANOVA. (C) CM from M2-polarized AMs, and to a lesser extent M0 AMs, enhanced circadian amplitude in *Per2*-Luc lung fibroblasts. Representative of *n* = 3. (D) M2 CM significantly increased circadian amplitude compared to untreated *Per2*-Luc fibroblasts. Data are represented as mean ± SEM. *n* = 3 biological repeats, ∗*p* < 0.05, one-way ANOVA.

Similarly, CM from *in vitro* polarized primary AMs was also added to *Per2*-Luc fibroblasts. CM from AM M0, M1, and M2 macrophages all promoted an increase in circadian amplitude (Figure 2C). A significant amplitude increase was observed exclusively with AM M2 macrophage CM (Figure 2D). These findings suggest that macrophage-derived soluble factors, particularly those released by M2-polarized macrophages, play a key role in enhancing circadian amplitude in fibroblasts.

### Macrophages increase collagen deposition when co-cultured with fibroblasts

To investigate the impact of macrophages on collagen deposition in fibroblasts, *in vitro* co-culture experiments were performed, followed by IF analysis to visualize and quantify changes in fibroblast-derived collagen. Co-cultures of polarized THP-1 macrophages and fibroblasts were seeded at a 1:5 ratio, as this is similar to the ratio between macrophages and fibroblasts in human lungs.^18^^,^^19^ Collagen deposition was visualized using confocal microscopy (Figure 3A), and collagen fibril area was quantified in these cultures with results normalized to nuclei numbers (Figure 3B). Lung fibroblasts co-cultured with M0-, M1-, or M2-polarized THP-1 macrophages all deposited significantly more collagen fibrils compared with fibroblasts cultured alone. Fibronectin staining in fibroblast-macrophage co-cultures revealed no changes in fibronectin deposition after the addition of polarized macrophages (Figure S2A). This finding confirms that the observed increase in collagen deposition is not due to a generalized upregulation of ECM components. To further explore whether this fibril-enhancing effect requires cell-cell contact or through soluble factors secreted by macrophages, CM from polarized THP-1 macrophages was added to fibroblast cultures (Figure 3C). Notably, only CM from M2-polarized macrophages significantly enhanced collagen deposition in fibroblasts compared with lung fibroblast cultures without CM (Figure 3D).

**Figure 3:**
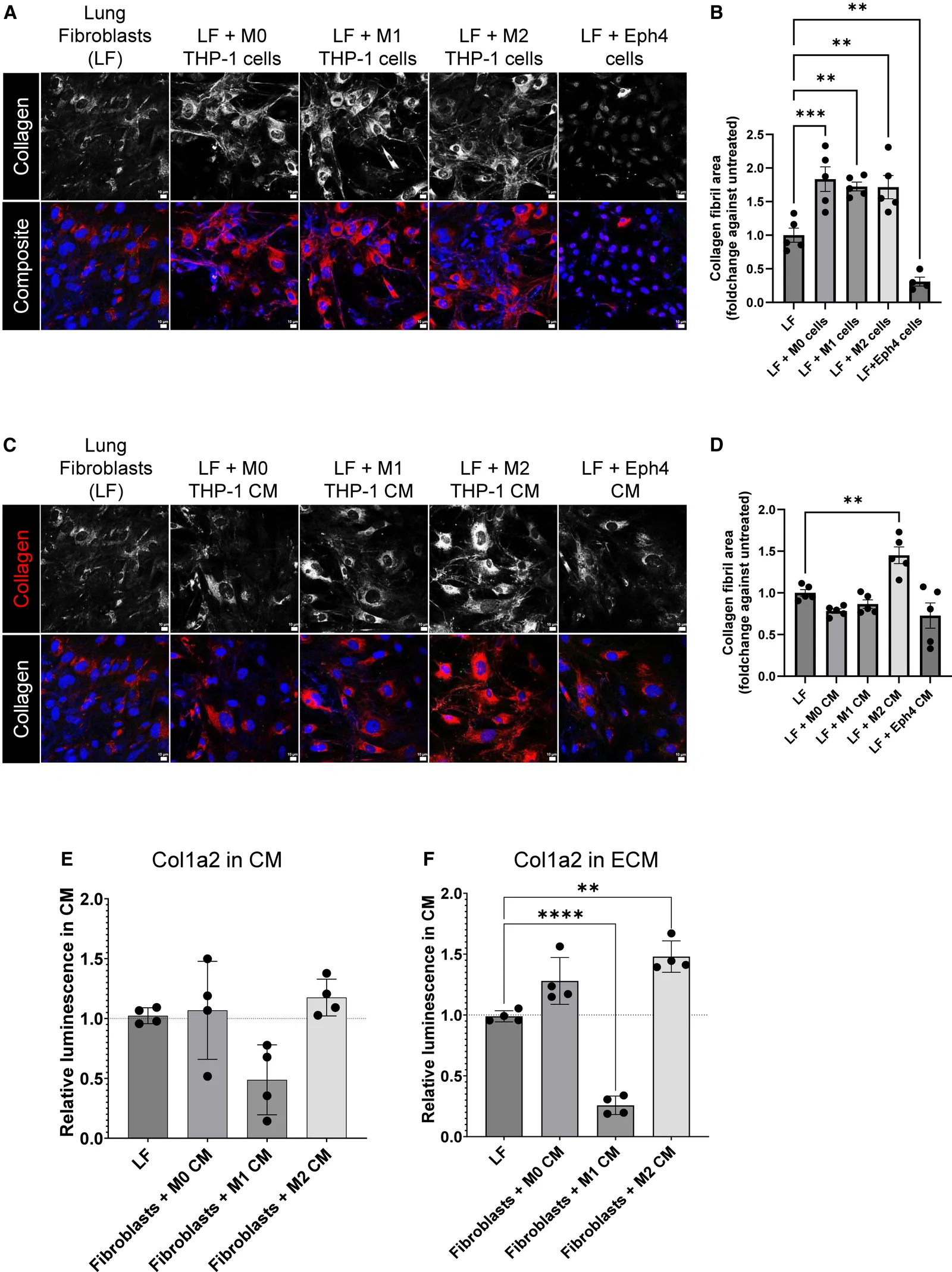
Quantification of collagen fibrils deposited by lung fibroblasts with or without THP-1 cells (A) IF images of LF and THP-1 macrophage co-cultures. Top images: collagen channel; bottom images: pseudo-colored collagen (red) and DAPI-stained nuclei (blue). Scale bars, 10 μm. Representative of *n* = 6. (B) Quantification of collagen fibril deposition in fibroblast cultures ± THP-1 macrophages. Data are represented as mean ± SEM. *n* = 6 biological repeats, ∗∗*p* < 0.01, ∗∗∗*p* < 0.005, one-way ANOVA. (C) IF images of fibroblasts with THP-1 macrophage-conditioned medium (CM). Layout similar to Figure 3A. Scale bars, 10 μm. Representative of *n* = 5. (D) Quantification of collagen fibril area ± macrophage CM. Data are represented as mean ± SEM. *n* = 5 biological repeats. ∗∗*p* < 0.01, one-way ANOVA. (E) Primary murine HiBiT-Col1a2 fibroblasts were treated with CM from polarized primary AMs for 4 days before replacing with fresh media. Luminescence was measured in the media of these cultures after 48 h. Luminescence is proportional to amount of Col1a2 in media. There was no significant difference in HiBiT luminescence readings between LF only and LF cultures treated with M0, M1, or M2 CM. Data are represented as mean ± SD. *n* = 4 biological repeats, one-way ANOVA. (F) Luminescence readings were also taken in the culture wells of LF samples to assess luminescence and therefore Col1a2 deposition into the matrix. M2 CM significantly increased, while M1 CM significantly decreased the deposition of fibroblast-derived tagged Col1a2 into the ECM. Data are represented as mean ± SD. *n* = 4 biological repeats, ∗∗*p* < 0.01, ∗∗∗∗*p* < 0.0001, one-way ANOVA.

Previously it was reported that macrophages can deposit collagen in mouse cardiac wound sites.^4^ Here, we cultured M0, M1, and M2 THP-1 macrophages at different cell numbers for 4 days in the presence of ascorbic acid. Only cultures with over 80% confluency deposited collagen fibrils (Figure S2B), indicating that while macrophages can produce collagen fibrils, it is unlikely that they are generating fibrils at the cell number used in our co-culture systems. To directly assess fibroblast specific collagen deposition, HiBiT-Col1a2 lung fibroblasts were used. These fibroblasts contain a genetically modified *Col1a2* gene, containing a bioluminescence-tagged collagen alpha-2(I) chain (protein schematic shown in Figure S2C). To confirm the HiBiT-tag was incorporated into collagen fibrils, HiBiT-Col1a2 fibroblasts were imaged and stained using both antibodies against HiBiT and collagen. IF imaging showed co-localization between HiBiT and collagen-I signals, indicating that the HiBiT-collagen is incorporated into fibrils as normal collagen (Figure S2D). HiBiT-Col1a2 fibroblasts were then cultured with macrophage CM for four days. Collagen deposition was quantified by measuring relative luminescence of two distinct sampling: (1) collagen released into the media (CM, i.e., secreted protomeric collagen) and (2) collagen deposited into the ECM (i.e., collagen fibrils) (Figures 3E and 3F). Luminescence readings of fibroblast CM showed no significant changes in collagen release when cultured with macrophages (Figure 3E). However, luminescence readings of collagen deposited into the ECM revealed that M2 CM significantly increased collagen luminescence readings, indicating enhanced collagen deposition (Figure 3F). M1 CM resulted in a significant reduction in ECM collagen deposition.

To determine if the increased collagen deposition was due to elevated expression of collagen genes and thus protein production in fibroblasts, *Col1a1* gene expression was quantified by qPCR following the addition of polarized THP-1 macrophages and CM. Neither macrophage cells nor CM influenced *Col1a1* expression in fibroblasts, suggesting that the increase in collagen deposition is not through transcriptional control (Figures S2E and S2F).

### Collagen deposition in fibroblasts is reliant on a functioning circadian clock

We have previously identified that collagen secretion and deposition in tendon fibroblasts are dependent on circadian rhythms.^5^ Here, to assess the impact of circadian dynamics on collagen fibril deposition in the lung, lung fibroblast cultures were analyzed by IF over a 24-h period at 4-h intervals. Collagen deposition was found to exhibit rhythmicity in both fibroblast monocultures and co-cultures with THP-1 macrophages (Figures 4A and 4B, respectively), whereby co-cultures had a larger collagen fibril area at the peak of fibril numbers, as demonstrated by representative images showing fewer collagen fibrils at post-synchronization (PS) time 35 compared with PS43 (Figure 4C).

**Figure 4:**
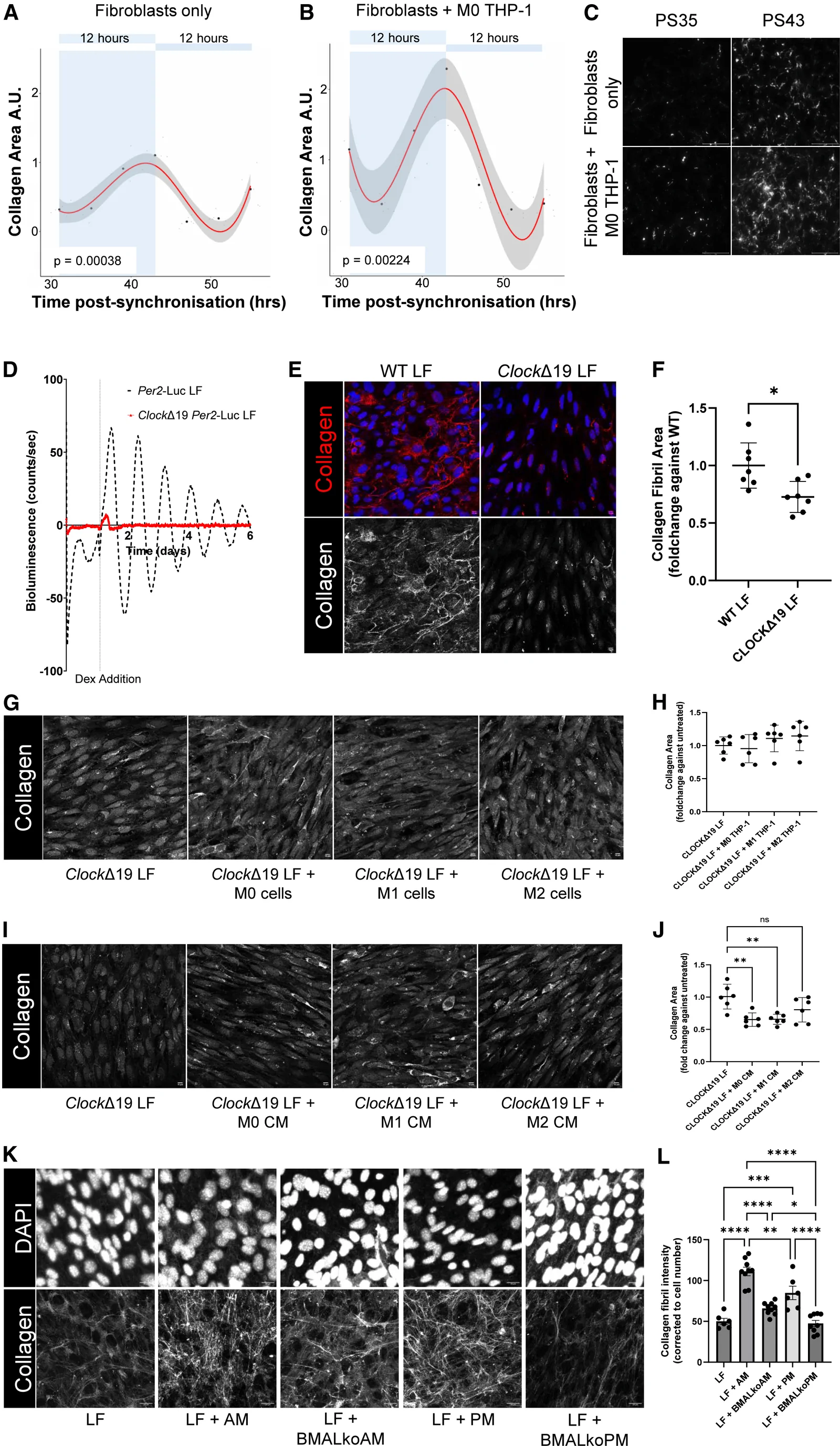
The relationship between circadian dynamics in lung fibroblasts and collagen deposition (A) Fibroblasts monoculture deposited collagen rhythmically over 24 h after synchronization with dexamethasone. Circadian analysis to determine rhythmicity was conducted using cosinor2 package in R to fit a cosinor model and assess rhythmicity. Representative of *n* = 3. *p* = 0.00038, *p* value calculated using cosinor.detect() function on R. (B) Fibroblast co-cultured with macrophages deposited collagen rhythmically over 24 h after synchronization with dexamethasone, and has a much higher collagen area compared to monoculture at peak time (PS43). Circadian analysis to determine rhythmicity was conducted using cosinor2 package in R to fit a cosinor model and assess rhythmicity. Representative of *n* = 3. *p* = 0.00224, *p* value calculated using cosinor.detect() function on R. (C) IF images of collagen fibrils at 35- and 43- hours post-synchronization (PS) in lung fibroblast (LF) monocultures and co-cultures with M0 THP-1 macrophages. Representative of *n* = 3. (D) Bioluminescence readings indicated dexamethasone initiated a rhythm in *Per2*-Luc WT fibroblasts but not in *Clock*Δ19 fibroblasts. Representative of *n* = 3. (E) IF staining of collagen in *Clock*Δ19 and WT fibroblast cultures showed collagen (red) and nuclei (blue) in pseudo-color images (top) and grayscale collagen-only images. Scale bars, 10 μm. Representative of *n* = 7. (F) Quantification of collagen deposition in *Clock*Δ19 LFs alone and WT LFs showed that *Clock*Δ19 fibroblasts deposit significantly less collagen than WT LFs. Data are represented as mean ± SEM. *n* = 7 biological repeats, ∗*p* < 0.05, unpaired two-tailed *t* test. (G) Representative IF images show *Clock*Δ19 fibroblasts alone and co-cultured with polarized macrophages. Scale bars, 10 μm. Representative of *n* = 6. (H) Quantification of collagen area showed that polarized macrophages did not increase collagen deposition in *Clock*Δ19 fibroblasts, with similar collagen levels observed in monocultures and co-cultures. Data are represented as mean ± SEM. *n* = 6 biological repeats, one-way ANOVA. (I) Representative IF images show *Clock*Δ19 fibroblasts alone and co-cultured with CM from polarized macrophages. Scale bars, 10 μm. Representative of *n* = 6. (J) CM from polarized macrophages did not increase collagen deposition in *Clock*Δ19 fibroblasts, in contrast M0 and M1 CM led to a decrease in collagen signal. Data are represented as mean ± SEM. *n* = 5 biological repeats for all bar *Clock*Δ19 fibroblasts alone (*n* = 6), ∗∗*p* < 0.01, one-way ANOVA. (K) Representative IF images show LFs in monoculture alone and co-cultured with primary alveolar macrophages (AMs) or peritoneal macrophages (PM) isolated from wildtype or Col6a1-Cre BMALko mice (BMALko). Top images: DAPI to show nuclei, bottom images: collagen-I staining. Scale bars, 20 μm. Representative of *n* = 4. (L) Quantification of collagen fibril intensity showed that primary macrophages (AM and PM) significantly increased collagen deposition in LFs, which was abrogated when primary macrophages were isolated from Col6a1-Dre BMALko mouse (BMALkoAM and BMALkoPM). Data are represented as mean ± SEM. More than 6 technical repeats across *n* = 4 biological repeats, ∗*p* < 0.05, ∗∗∗*p* < 0.005, ∗∗∗∗*p* < 0.001, one-way ANOVA.

To further explore the role of the circadian clock in this process, fibroblasts with a mutated *Clock* gene (*Clock*Δ19, which results in a 51-amino acid deletion at C-terminal of the CLOCK protein and thus impaired circadian rhythm) were evaluated. Bioluminescence recordings showed negligible rhythmic activity apart from an initial peak following dexamethasone synchronization (Figure 4D). Addition of macrophages did not rescue the rhythm in these *Per2*-Luc *Clock*Δ19 fibroblasts (Figure S3A).

Collagen deposition in *Clock*Δ19 fibroblasts was compared with WT fibroblasts. IF analysis revealed significantly reduced collagen deposition in *Clock*Δ19 lung fibroblast cultures (Figures 4E and 4F), suggesting a critical role for an intact fibroblast circadian clock in regulating collagen synthesis and ECM dynamics. To determine whether macrophages could compensate for the impaired circadian regulation of collagen deposition in *Clock*Δ19 fibroblasts, these cells were co-cultured with polarized THP-1 macrophages or treated with macrophage CM. IF images and quantification demonstrated that co-cultures with M0, M1, or M2 macrophages did not rescue collagen deposition in *Clock*Δ19 fibroblasts (Figures 4G and 4H). Interestingly, the addition of M0, M1, or M2 macrophage CM to *Clock*Δ19 fibroblasts appeared to reduce collagen signals in clockless fibroblasts (Figures 4I and 4J). Together, these findings highlight a reliance of fibroblasts on an intact circadian clock for ECM remodeling. The involvement of circadian rhythms was further confirmed when fibroblast-macrophage co-culture was treated with the small molecule Cry1/Cry2 stabilizer KL001,^20^ where 5 uM treatment, which reduced circadian rhythms (Figure S3B), also significantly inhibited macrophage-induced collagen deposition (Figures S3C and S3D). Of note, when macrophages isolated from Col6a1-Cre BMALfl/fl mouse,^21^ where circadian rhythms are either abolished or dampened (Figures S3E and S3F), were co-cultured with normal lung fibroblasts, the collagen-inducing effects were also significantly reduced to baseline monoculture level (Figures 4K and 4L). This highlights that a functional circadian rhythm is required in both fibroblasts and macrophages for collagen deposition. However, it should be noted that not all effects on collagen deposition by fibroblasts require an increase in circadian rhythm, for example in the case of treatment. Here, treatment with TGFβ1 (5 ng/mL) increased collagen-I deposition without affecting circadian rhythms (Figures S3G and S3H).

### PDGFA is highly upregulated in M2 conditioned media

As M2 CM induces the most robust circadian rhythm and enhances collagen deposition in fibroblasts, we aimed to identify the factor(s) responsible for this change. CM from M0, M1, and M2 THP-1 macrophages were analyzed by MS to identify proteins significantly upregulated in M2 CM compared with M0 and M1 CM. Proteins identified through MS were clustered using *k*-means clustering, resulting in two distinct clusters. Proteins were considered significantly differentially expressed if they exhibited a fold-change (FC) ≥ 2 and a *p* value ≤ 0.05.

Cluster 1 comprised of proteins upregulated in M1 CM compared with M0 and M2 CM (Figure 5A). Established known M1-expressed proteins in this cluster include CXCL8 and CXCL10,^22^ which were among the most differentially expressed genes compared to M2 CM, confirming the polarization status of the macrophages. Cluster 2 contained proteins upregulated in M2 CM, with M2 macrophages showing greater proteomic similarity to M0 than to M1 (Figure 5B). Among the proteins elevated in M2 CM, PDGFA emerged as the most highly differentially expressed, displaying strongly positive log2 FC values when compared to both M0 and M1 CM.

**Figure 5:**
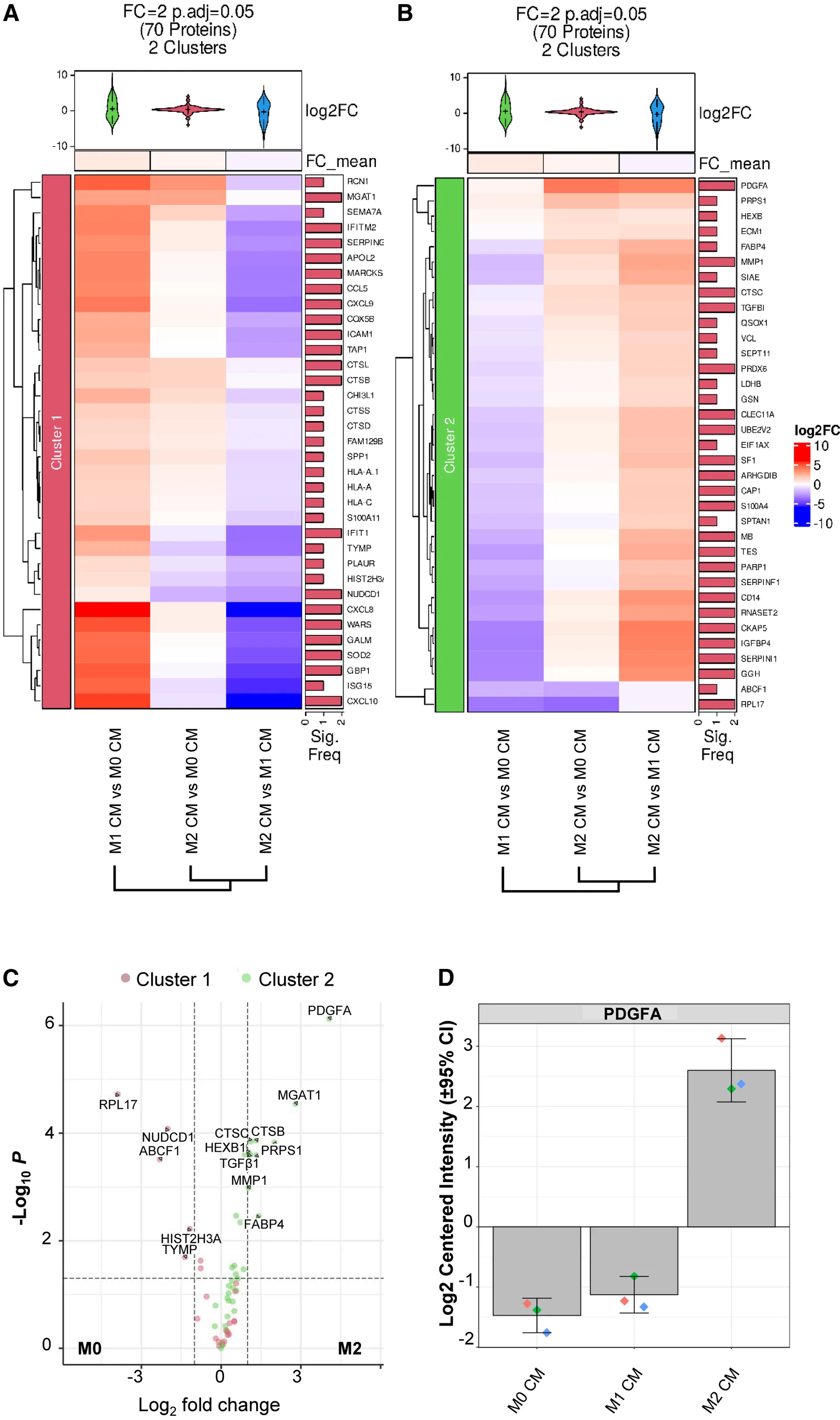
Comparative proteomic analysis of differentially expressed proteins in M0, M1, and M2 AM CM (A) Heatmap analysis of differentially expressed proteins in M0, M1, and M2 AM CM, based on MS data processed with the Manchester Proteome Profiler. Fold change values (≥ 2, *p* ≤ 0.05) were clustered using *k*-means. Cluster 1 highlights proteins upregulated in M1 CM, while cluster 2 shows proteins upregulated in M2 CM. *n* = 3 biological repeats. (B) Cluster 2 includes PDGFA, which is highly expressed in M2 CM but not M0 or M1 CM. *n* = 3 biological repeats. (C) Volcano plot of proteins in M2 CM vs. M0 CM. Proteins significantly upregulated in M0 CM appear on the left, while those upregulated in M2 CM, including PDGFA as the most prominent, appear on the right. *n* = 3 biological repeats. (D) Barplot showing positive log2 centered PDGFA intensity in M2 CM samples only. *n* = 3 biological repeats.

To visualize relative protein expression, volcano plots were generated using the Manchester Proteome Profiler. These plots applied the same FC and *p* value thresholds, with dashed lines marking the cut-offs. Proteins differentially expressed between M2 and M0 CM are highlighted in Figure 5C, where green dots indicate proteins elevated in M2 CM and pink dots represent those higher in M0 CM. PDGFA appeared prominently in the top-right quadrant, signifying significant upregulation in M2 CM. Additional analysis comparing M2 to M1 CM, and M1 to M0 CM, was performed (Figures S4A and S4B).

The abundance of PDGFA in M2 CM was further emphasized when the data are presented as log2-centered intensity (Figure 5D), where positivity was exclusively observed in M2 CM samples. These findings highlight PDGFA as a key M2 macrophage-derived factor that may be driving the observed effects on fibroblast circadian dynamics and collagen deposition.

As PDGFA has relatively high thermal stability,^23^ CM from THP-1 macrophages was also subjected to heat-treatment. The medium was heated to 95°C for 30 min, cooled to room temperature for 30 min, and then added to *Per2*-Luc lung fibroblasts. Heat-treated CM induced a circadian rhythm in the fibroblasts similar to that of untreated CM. Quantification of circadian amplitude revealed no significant differences between heat-treated and untreated CM (Figures S4C and S4D), confirming heat stability in the rhythm-inducing soluble factor(s) and providing further evidence that PDFA is likely involved in driving circadian changes in fibroblast cultures. Of note, prediction of transcription factor binding using ConTra v3^24^ identifies an E-box element within 500 bp of the promoter region of PDGFA, suggesting that PDGFA itself may be under circadian control.

### Macrophage-derived PDGFA enhances collagen deposition and increases circadian rhythm amplitude in fibroblasts

PDGFA was investigated as a candidate protein responsible for enhancing collagen deposition and circadian rhythm amplitude in fibroblasts. We first approached this using recombinant PDGFA at a concentration of 17.5 ng/mL, consistent with what has been measured in whole blood serum.^25^ Addition of recombinant PDGFA to *Per2*-Luc fibroblasts significantly increased circadian amplitude (Figures 6A and 6B). We then performed CRISPR on THP-1 cells to knockout (KO) PDGFA expression (PDGFAko). The KO was confirmed by sequencing and western blot (Figures S5A and S5B). Furthermore, unlike WT THP-1 macrophages, PDGFAko THP-1 did not induce a sustained rhythm in *Per2*-Luc fibroblasts (Figures 6C and 6D).

**Figure 6:**
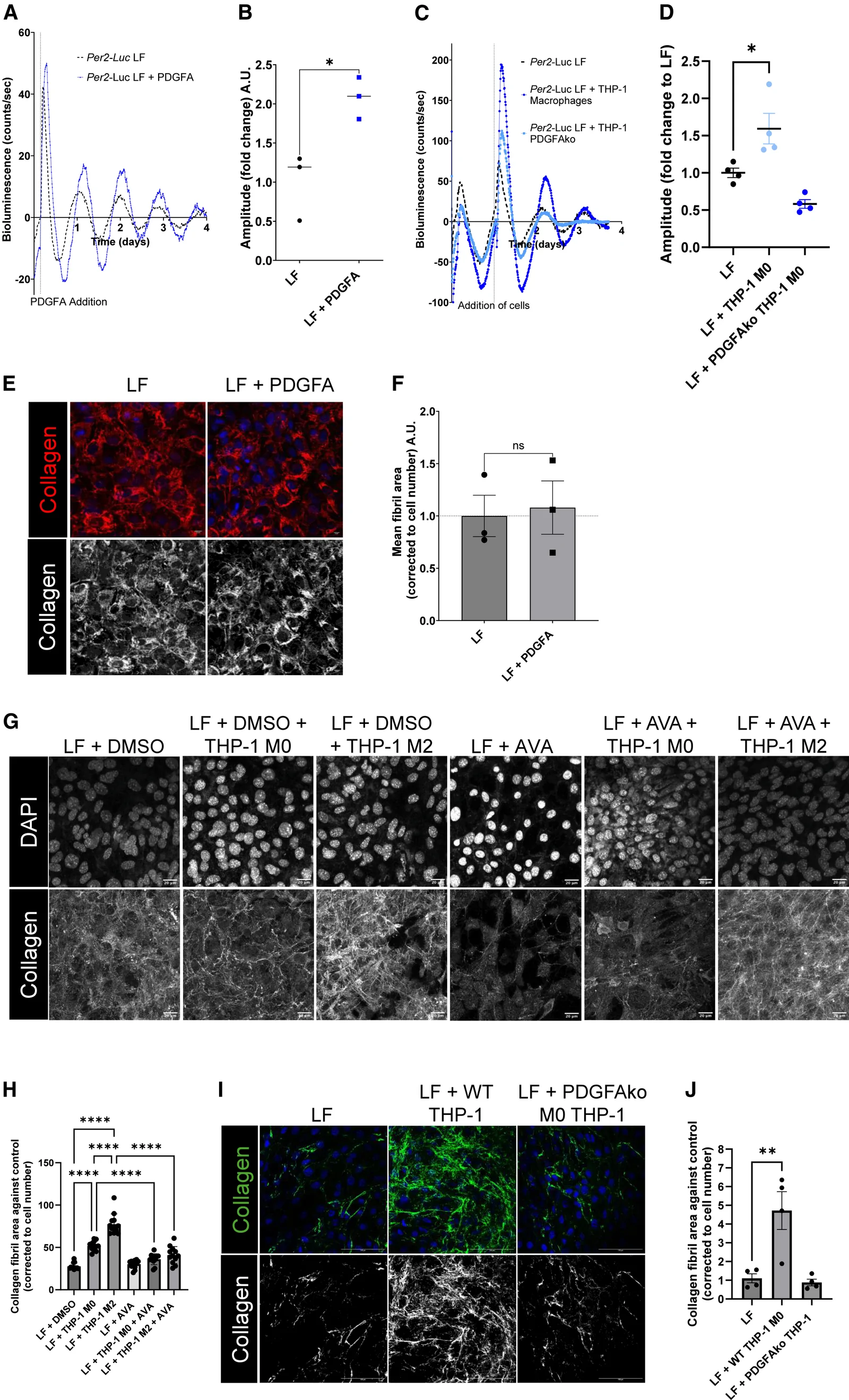
Evaluation of PDGFA’s capability to enhance circadian amplitude and collagen deposition in fibroblasts (A) Recombinant PDGFA induced a robust circadian rhythm in *Per2*-Luc lung fibroblasts (LFs). Representative of *n* = 3. (B) Amplitude fold change analysis demonstrated that PDGFA significantly enhances circadian amplitude. Data are represented as mean ± SEM. *n* = 3 biological repeats, ∗*p* < 0.05, unpaired two-tailed *t* test. (C) THP-1 PDGFAko failed to induce a sustained and robust circadian rhythm in *Per2*-Luc LFs. Representative of *n* = 4. (D) Amplitude fold change analysis showed that WT THP-1 M0 significantly increased circadian amplitude in fibroblasts, while PDGFAko THP-1 M0 did not. Data are represented as mean ± SEM. *n* = 4 biological repeats, ∗*p* < 0.05, on-way ANOVA. (E) IF images of LFin with recombinant PDGFA. Top images show composite images of DAPI-stained nuclei (blue) and collagen (red), while bottom images show the collagen channel only. Representative of *n* = 3 biological repeats. Scale bars, 10 μm. (F) Quantification revealed there was no statistical difference between collagen deposition in fibroblast cultures following incubation with PDGFA. Data are represented as mean ± SEM. *n* = 3 biological repeats, unpaired two-tailed *t* test. (G) IF images of LFs in monoculture and co-culture with M0/M2 THP-1 macrophages, either in presence of 100 nM avapritinib (AVA) or 0.1% DMSO vehicle control. Top images shows DAPI staining for nuclei, bottom images shows collagen signal. Representative of *n* = 3 biological repeats. Scale bars, 20 μm. (H) Quantification of collagen fibril area showed that treatment with AVA abrogates any macrophage-induced collagen deposition increase. Data are represented as mean ± SEM. 12 technical repeats across *n* = 3 biological repeats. ∗∗∗∗*p* < 0.0001, one-way ANOVA. (I) IF images of LFs in monoculture and co-culture with either PDGFA KO THP-1 or WT THP-1 macrophages. Top images show composite images of DAPI-stained nuclei (blue) and collagen (green), while bottom images show the collagen channel only. Representative of *n* = 4. Scale bars, 100 μm. (J) Quantification revealed that WT THP-1 macrophages significantly increased collagen deposition in fibroblasts, whereas PDGFA KO THP-1 macrophages did not. Data are represented as mean ± SEM. *n* = 4 biological repeats, ∗∗*p* < 0.005, one-way ANOVA.

To assess whether recombinant PDGFA alone could stimulate collagen deposition in fibroblast cultures, imaging studies were conducted. As shown in Figure 6E, no significant difference in collagen deposition was observed when fibroblasts were treated with recombinant PDGFA compared to untreated controls. Quantitative analysis of collagen area further confirmed that PDGFA alone did not influence collagen deposition in fibroblasts (Figure 6F). We considered that the concentration of recombinant PDGFA used was not sufficient to induce collagen fibrillogenesis effects, and performed a dose-response experiment to investigate the effects of higher PDGFA concentrations (8.75 ng/mL to 140 ng/mL) on fibroblast circadian rhythm and collagen deposition. Interestingly, most PDGFA doses showed a trend of activating fibroblasts circadian rhythms, although only 17.5 ng/mL was significant (Figures S5C and S5D) and no dose-dependent response was observed. In contrast, recombinant PDGFA did not significantly increase collagen fibril area in all doses tested (Figure S5E).

The effects of PDGFA signaling on fibroblast collagen deposition as well as circadian regulation were further tested using the PDGFRa specific inhibitor avapritinib (AVA).^26^ Here, we performed a co-culture experiment with M0 and M2 THP-1-derived macrophages in the presence of 100 nM AVA or vehicle (0.1% DMSO), and found that AVA treatment led to a significant reduction in macrophage-induced collagen deposition (Figures 6G and 6H). Interestingly, AVA treatment also abrogated fibroblast circadian rhythms; however, this is not due to fibroblast cell death, as AVA treatment after 72 h did not induce a significant change in cell numbers, apart from an increase in AVA treatment in fibroblast-M0 co-culture (Figures S5F and S5G). To investigate how PDGFA signaling affects circadian rhythms, qPCR against core clock genes was carried out. Recombinant PDGFA treatment had no significant effects on core clock gene mRNA expression compared to untreated fibroblasts (Figure S5H); however, interestingly, fibroblasts co-cultured with macrophages have elevated BMAL and CLOCK mRNA levels, which were abrogated with AVA treatment (Figure S5I). This suggests that, specifically, macrophage-induced PDGFRa signaling causes circadian changes at the transcriptional level.

IF imaging was also carried out to compare the effects of PDGFAko and WT THP-1 macrophages on collagen deposition (Figure 6I). Consistent with previous findings, fibroblasts co-cultured with WT THP1 deposited a significantly increased amount of collagen fibrils when compared with fibroblast monoculture (middle images vs. left images), whereas fibroblasts co-cultured with PDGFAko THP-1 macrophages have similar levels of collagen fibrils compared with monoculture (right images vs. left images), as confirmed by quantification (Figure 6J). This indicates that macrophage-derived PDGFA drives increase of collagen fibrillogenesis.

Together, these results identify macrophage-derived PDGFA/PDGFRa signaling as a key factor that enhances collagen deposition and circadian rhythm amplitude in fibroblasts, highlighting it as a mediator of macrophage-fibroblast crosstalk.

## Discussion

In this study, we provided evidence that polarized macrophages, as well as soluble factors secreted by M2-polarized macrophages, promote collagen deposition in fibroblasts via a clock-mediated mechanism. These results highlight the importance of effective crosstalk between macrophages and fibroblasts, as well as demonstrating the necessity of a functioning clock, to maintain a functional ECM. These data therefore have implications for tissue remodeling and repair processes. It is important to note that, in general, “M2-polarized macrophages” refers to multiple different subtypes with certain features in common, such as being anti-inflammatory, but the specific roles and characteristics depend on the cell’s exposure to different cytokines and growth factors, etc. In this study, M2-polarized macrophages refers only to naive M0 macrophages polarized with either IL-4c (*in vivo*) or IL-4 and IL-13 (*in vitro*).

While M0, M1, and M2 THP-1 macrophages all increased collagen deposition and circadian amplitude in co-cultures with fibroblasts, only CM from M2-polarized macrophages had the same effect. This is consistent with M2 macrophages’ phenotype, which involves ECM remodeling and pro-wound-healing characteristics.^27^^,^^28^ These findings therefore suggest that macrophages regulate fibroblast function via both paracrine and juxtacrine signaling pathways. However, M2-polarized macrophages have an increased capacity to regulate collagen deposition in fibroblasts, via elevated secretion of circadian-modulating soluble factors—a mechanism that would allow M2 macrophages to drive ECM remodeling.

We have previously identified that fibroblasts utilize endocytic recycling as a mechanism for collagen fibrillogenesis,^29^ and prior research has identified that macrophages can directly deposit collagen during heart repair.^4^ These observations suggest that the source of collagen in a complex environment may not be strictly from fibroblast-like cells. Here we observed that macrophages, regardless of polarization status, can produce collagen fibrils, although morphologically different from fibroblast-derived fibrils and only observed at high cell density. As the number of macrophages in our co-culture systems was much lower than fibroblasts, and macrophages are terminally differentiated cells that do not replicate *in vitro*, it is highly likely that the collagen fibrils are produced by fibroblasts only, although we cannot rule out that macrophage-derived collagen may have been taken up by fibroblasts and utilized for fibrillogenesis. This could be addressed in the future by utilizing an endogenous collagen-specific HiBiT tag in macrophages and co-culturing with fibroblasts. The bioluminescence signal could then be used to determine the amount of collagen within the ECM derived from macrophages.

*In vitro* polarized M0 and M2 AMs both enhanced circadian amplitude in fibroblasts, whereas only *in vivo* polarized M2 macrophages induced a similar effect. These differences may stem from the inherent variability of primary macrophages, influenced by their origin and microenvironment. Nevertheless, M2 macrophages consistently demonstrated a significant ability to induce circadian rhythms in fibroblasts, highlighting their heightened capacity for intercellular communication and modulation of fibroblast circadian dynamics. In the current study, we demonstrated that lung fibroblasts, like tendon fibroblasts, deposited collagen fibrils in a circadian rhythmic manner; further, clockless fibroblasts exhibited disrupted collagen deposition in both instances. However, the effect of knocking out circadian rhythm on collagen deposition is likely dependent on which tissue the fibroblast originated from, as we previously observed clockless tendon fibroblasts deposit more but thinner fibrils,^5^ whereas here the clockless lung fibroblasts had a severely diminished capability of collagen fibrillogenesis. We have also shown that a functional clock is required in both macrophages and fibroblasts to drive the collagen fibrillogenesis effects; however, the circadian status (i.e., synchronised/unsynchronized, ascending/descending circadian phase) did not seem to play a role here, as long as circadian rhythm can be modulated.

We observed that M2-secreted factors in particular significantly induced fibroblast circadian- and collagen-response. MS analysis on the secretome of M0, M1, and M2 macrophages identified over 1000 proteins, which were further filtered for decoys and known contaminants, and analyzed in a pairwise manner (M0 vs. M2, M1 vs. M2) to identify 35 of the most significantly differentially expressed (i.e., most upregulated and most down-regulated) proteins for each comparison. PDGFA was identified as the top upregulated protein in M2 CM compared to M0 CM and M1 CM, suggestive that it is a key macrophage-secreted factor that drives increased collagen deposition through a clock-dependent mechanism. Additional support for this hypothesis comes from the observation that heat treatment of M2 CM did not reduce its ability to enhance circadian amplitude in fibroblasts, consistent with the known thermal stability of PDGFA.^23^ In our studies, recombinant PDGFA significantly enhanced circadian amplitude in fibroblasts. Furthermore, while WT THP-1 macrophages increased circadian amplitude in fibroblasts, THP-1 macrophages lacking PDGFA (PDGFAko) failed to produce the same effect. PDGFRa-specific inhibitor avatinib can also inhibit circadian rhythms in fibroblasts, demonstrating that PDGFA signaling is essential for enhancing circadian amplitudes in co-culture systems.

Although PDGFA was found to be highly upregulated exclusively in CM from M2-polarized macrophages, unpolarized PDGFAko THP-1 macrophages were unable to enhance collagen deposition in fibroblasts, unlike their WT counterparts. This suggests that even low levels of PDGFA may be essential for facilitating effective cell-cell interactions between macrophages of all phenotypes and fibroblasts. This is supported by our PDGFRa inhibitor studies, which abrogated the collagen induction responses elicited by both M0 and M2 macrophages, confirming the relationship between macrophage-derived PDGFA and collagen deposition. Supporting this hypothesis, our data demonstrate that both M0 and M1 macrophages require direct cell contact to increase collagen fibril deposition in fibroblasts. This aligns with a previous study showing that PDGFA functions as a potent chemoattractant for fibroblasts.^30^ In contrast, CM from M2 macrophages alone is sufficient to promote collagen fibril deposition. Notably, however, recombinant PDGFA alone, regardless of dose used (up to 8× the level detected in serum), was unable to increase collagen deposition in fibroblasts. This is an unexpected finding, as previous data have shown that mice with a PDGFRα KO exhibited significantly reduced collagen deposition, demonstrating that PDGFA signaling plays a crucial role in collagen regulation.^31^ The disparity in findings may be indicative of the complexity of PDGF biology (e.g., dimerization partners, long/short isoforms of PDGFA^32^) which may not be reflected in the recombinant proteins. Further, macrophage-derived PDGFA may be involved in regulation of other M2-derived secreted factors that regulate fibroblast collagen deposition response.

Together, this indicates that recombinant PDGFA synchronizes the circadian clock in fibroblasts, but on its own is not sufficient to increase collagen deposition in fibroblasts, suggestive of a requirement for other macrophage-derived factors working in tandem. These may be other secreted cytokines, chemokines,^33^ or indeed microRNA (miRNA) and metabolites,^34^^,^^35^ that may be working in tandem with PDGFA to elicit both circadian and collagen responses.^36^^,^^37^ The fact that PDGFA was predicted to have an E-box binding site in its promoter region, coupled with the observation that PDGFRa inhibition led to a loss of circadian rhythm in fibroblasts, suggests that PDGFA signaling plays a more important role in regulating circadian-collagen deposition by fibroblasts than previously anticipated. However, it should be noted that while avapritinib is a highly specific inhibitor to PDGFRa, it may still have off-target effects (e.g., KIT), which cannot be ruled out in this present study. Future studies should knock out PDGFRa specifically in fibroblasts to verify the findings presented here. Further, as macrophages’ own synchronicity is not required to induce the circadian/collagen effects seen in fibroblasts, this suggests that macrophages secrete robust synchronizing signals such as PDGFA, that reset the circadian clock of fibroblasts at any time of day. This may also explain why loss of macrophage circadian rhythm no longer induces collagen deposition, as this will likely influence PDGFA expression.

As living organisms generally have their circadian rhythms entrained daily, a question is the relevance of our findings here at the *in vivo* level, i.e., in what circumstances would fibroblast lose its synchronicity. This was hinted by our *in vitro* wounding assay, where post-wound fibroblast circadian rhythms were disrupted. Other studies have demonstrated that pathological conditions such as fibrosis and cancer also have disrupted circadian rhythms.^9^^,^^38^^,^^39^ One may postulate that at disease initiation stage there may already be low levels of circadian disruptions which the immune system tries to “correct”, and that perhaps the prolonged inability of the circadian correction then drives disease progression. How the maintenance of a circadian rhythm by immune infiltration happens *in vivo*, and how this relates to tissue homeostasis and disease, is another important future research direction. Nonetheless, our findings in this study point to a crucial role for PDGFA/PDGFRa in synchronizing fibroblast circadian rhythms, which may subsequently influence the temporal regulation of CCGs involved in the collagen secretory pathway. Although beyond the scope of the present study, future studies should aim to identify additional components of M2 CM that work with PDGFA to augment fibroblast collagen deposition. These efforts could involve proteomic and transcriptomic analyses to characterize the secretome of M2 macrophages, with or without PDGFA, comprehensively.

An alternative, but not mutually exclusive, explanation is that PDGFA-mediated circadian synchronization enhances the responsiveness of fibroblasts to other macrophage-derived factors. The circadian clock regulates the expression of genes involved in the collagen secretory pathway.^5^ By amplifying the circadian amplitude, PDGFA may prime fibroblasts for optimal temporal coordination of these CCGs, thereby facilitating increased collagen deposition when additional signals are present. Of note, a mix of male and female mice was used in this study for primary cell isolation. The sexes were kept separate and we did not observe overt differences between them in terms of collagen or circadian responses. However, as mentioned previously, 2D *in vitro* assays were used in this study, and one cannot exclude an influence of sexes in more complex 3D/*ex vivo*/*in vivo* systems.

Collectively, our findings suggest that M2 macrophages secrete multiple factors that drive fibroblast circadian synchronization and subsequent collagen deposition. While PDGFA is a key mediator, the presence of additional soluble factors is likely critical to the observed effects. Further studies are warranted to identify and characterize these factors and to elucidate their individual and collective contributions to circadian regulation and ECM remodeling. Insights from these studies could guide the development of therapeutic strategies aimed at modulating macrophage-fibroblast interactions to optimize tissue repair mechanisms.

### Limitations of the study

This study has shown that macrophages modulate fibroblasts and subsequent collagen deposition responses, implicating a role in normal-wound healing response and identified PDGFA/PDGFRa as a key signaling axis. One caveat is that the studies were conducted only in *in vitro* systems and should be confirmed *in vivo*. Further, whether PDGFA signaling is also circadian-dependent and/or how this controls fibroblast circadian rhythms remains unknown. Additionally, here we demonstrated the directional control between macrophages and fibroblasts in lung fibroblasts; however, this response may be tissue-dependent.

## Resource availability

### Lead contact

Further information and requests for resources and reagents should be directed to and will be fulfilled by the lead contact, Joan Chang (joan.chang@manchester.ac.uk).

### Materials availability

Cell lines generated in this study will be made available on request to the lead contact.

### Data and code availability

• The mass spectrometry proteomics data have been deposited to the ProteomeXchange Consortium via the PRIDE partner repository as listed in the key resources table. Original western blot images and microscopy data reported in this paper will be shared by the lead contact upon request.
• This paper does not report original code.
• Any additional information required to reanalyze the data reported in this paper is available from the lead contact upon request.

## Acknowledgments

The proteomics was performed at the Biological Mass Spectrometry Facility with the assistance of Stacey Warwood, imaging was performed at the Bioimaging Facility, the PDGFA knockout line was generated by W.-S.L. within the Genome Editing Unit (GEU); all facilities are in the Faculty of Biology, Medicine and Health (University of Manchester), and supported by funding from Wellcome, BBSRC, and the University of Manchester Strategic Fund. We thank the University of Manchester Biological Support Facility for expertize and assistance in animal welfare and husbandry. This study is supported by MRC Career Development Award MR/W016796/1 (to J.C.), Wellcome Trust Immunomatrix in Complex Disease (ICD) PhD studentship 218491/Z/19/Z (to K.L.), BBSRC strategic Longer and Larger grants BB/T001984/1 (to M.F.A.C., Q.-J.M., and K.E.K.), and Wellcome 226804/Z/22/Z (to J.C., Q.-J.M., GEU).

## Author contributions

J.C., T.H., and K.E.K. conceived the project. J.C., K.E.K., Q.-J.M., T.H., and A.S.M. supervised the experiments. K.L., X.-Y.Z., X.-F.Y., M.F.A.C., S.-Y.L., J.C., and M.C. performed experiments and interpreted data. J.J.H. performed *in vivo* polarization experiments. J.K. performed proteomics experiments and analysis. A.S.M., Q.-J.M., and S.-Y.L. provided reagents and data interpretation. All authors contributed to writing the manuscript.

## Declaration of interests

The authors declare no competing interests.

## STAR★Methods

### Key resources table

**Table:** 

REAGENT or RESOURCE	SOURCE	IDENTIFIER
**Antibodies**		

Anti-Collagen Alpha-1(I) Chain Carboxy-Telopeptide [LF-68] Antibody	Kerafast	Cat#ENH018-FP, RRID:AB_3741809
Anti-Fibronectin Antibody	Merck Sigma Aldrich	Cat#F6140, RRID:AB_476981
Anti-HiBiT Monoclonal Antibody	Promega	Cat# N7200, RRID:AB_3665694
Goat anti-Mouse IgG (H + L) Cross-Adsorbed Secondary Antibody, Alexa Fluor™ 488 - A-11001	Thermo Fisher Scientific	Cat# A-11001, RRID:AB_2534069
Goat anti-Rabbit IgG (H + L) Cross-Adsorbed Secondary Antibody, Alexa Fluor™ 488 - A-11008	Thermo Fisher Scientific	Cat# A-11008, RRID:AB_143165
Goat anti-Mouse IgG (H + L) Cross-Adsorbed Secondary Antibody, Alexa Fluor™ 647 - A-21235	Thermo Fisher Scientific	Cat# A-21235, RRID:AB_2535804
Goat anti-Rabbit IgG (H + L) Cross-Adsorbed Secondary Antibody, Alexa Fluor™ 647 - A-21244	Thermo Fisher Scientific	Cat# A-21244, RRID:AB_2535812
Goat anti-Rabbit IgG (H + L) Cross-Adsorbed Secondary Antibody, Cyanine3 - A10520	Thermo Fisher Scientific	Cat# A10520, RRID:AB_10563288
Goat anti-Mouse IgG (H + L) Cross-Adsorbed Secondary Antibody, Cyanine3 - A10521	Thermo Fisher Scientific	Cat# A10521, RRID:AB_10373848
Goat anti-Rabbit IgG (H + L) Cross-Adsorbed Secondary Antibody, Cyanine5 - A10523	Thermo Fisher Scientific	Cat# A10523, RRID:AB_2534032
BD Pharmingen™ Purified Rat Anti-Mouse IL-4	BD Biosciences	Cat# 554387, RRID:AB_398545
Anti-Platelet-derived growth factor subunit A (PDGFA) Antibody	RayBiotech	Cat#130–10142–20
Goat anti-Mouse IgG (H + L) Highly Cross-Adsorbed Secondary Antibody, Alexa Fluor™ 680	Thermo Fisher Scientific	Cat# A-21058, RRID:AB_2535724

**Bacterial and virus strains**		

per2-bre-luc2-ef1a-GFP vector	Qing Jun Meng lab	N/A
psPAX2 packaging vector	Addgene	RRID:Addgene_12260
pMD2.G envelope vector	Addgene	RRID:Addgene_12259

**Chemicals, peptides, and recombinant proteins**		

phorbol 12-myristate 13-acetate	Sigma	Cat#P8139
Mouse IL-4 Recombinant Protein, PeproTech®	Thermo Fisher Scientific	Cat#214–14
Lipopolysaccharides from *Escherichia coli* O55:B5	Merck Sigma Aldrich	Cat#297–473–0
Collagenase, Type IV, powder	Thermo Fisher Scientific	Cat#17104019
Human TGF-beta 1 Recombinant Protein, PeproTech® - 100-21-10UG	Thermo Fisher Scientific	Cat#100–21
Avapritinib	Cambridge Bioscience	Cat#HY-101561
KL001	SLS	Cat#SML1032
Human IFN-γ1b	Miltenyi Biotec	Cat#130–096–481
Human IL4	Miltenyi Biotec	Cat#130–093–920
Human IL13	Miltenyi Biotec	Cat#130–112–409
polybrene	Sigma Aldrich	Cat#TR-1003
Alt-R™ S.p. Cas9 Nuclease V3	IDT	Cat#1081058
Alt-R™ CRISPR Cas9 tracrRNA	IDT	Cat#1072532
Dexamethasone	SLS	Cat#D1756
TRIzol™ Reagent	Invitrogen	Cat#15596026

**Critical commercial assays**		

Nano-Glo® HiBiT Extracellular Detection Kit	Promega	Cat#N2420
TaqMan™ Reverse Transcription Reagents	Invitrogen	Cat#N8080234
SensiFAST™SYBR® No-ROX kit	Bioline	Cat#BIO-98005

**Deposited data**		

Thp1 conditioned media mass spectrometry proteomics analysis	This paper	ProteomeXchange PXD061123

**Experimental models: Cell lines**		

Human: Thp1	This paper	N/A
Human: HEK293T	This paper	N/A
Mouse: immortalized Per2:luc lung fibroblasts	This paper	N/A
Mouse: immortalized Clockd19 Per2:luc lung fiborblasts	This paper	N/A

**Experimental models: Organisms/strains**		

Mouse: PER2:Luciferase	This paper	RRID:IMSR_JAX:006852
Mouse: Col6a1-Cre BMALfl/fl	This paper	N/A

**Oligonucleotides**		

See Table S1 for list of oligonucleotides	Sigma Aldrich	N/A

**Software and algorithms**		

Fiji ImageJ	https://imagej.net/software/fiji/	https://doi.org/10.1038/nmeth.2019
BioDare2	https://biodare2.ed.ac.uk/	https://doi.org/10.1007/978-1-0716-1912-4_2
MetaMorph	https://www.moleculardevices.com/products/cellular-imaging-systems/high-content-analysis/metamorph-microscopy	N/A
GraphPad Prism 10.2.2	https://www.graphpad.com/scientific-software/prism/	N/A
MaxQuant 2.4.10.0	https://maxquant.org/	https://doi.org/10.1038/nbt.1511
Manchester Proteome Profiler	https://mpp.sbs.manchester.ac.uk/	https://doi.org/10.64898/2026.05.14.725092
Contra v3	http://bioit2.irc.ugent.be/contra/v3	https://doi.org/10.1093/nar/gkx376

### Experimental model and study participant details

#### Mouse studies

The care and use of all mice was carried out in accordance with the UK Home Office regulations, UK Animals (Scientific Procedures) Act of 1986 under the Home Office License numbers, S1-P57F83603, P44492AC9, PP7202625.

All lung fibroblasts (LFs) and alveolar macrophages (AMs) were isolated from C57BL/6J mice of various genotypes, aged between 2–3 months and of mixed sexes. *Per2*-Luc lung fibroblasts were derived from C57BL/6J mice bred on a background with PER2:Luciferase reporter.^14^ *Clock*Δ*19* mice (C57BL/6 on a *Per2*:Luciferase reporter background) were as described previously.^40^ Col6a1-Cre BMALfl/fl mice (C57BL/6 on a *Per2*::Luciferase reporter background) were as described previously.^21^

#### *In vivo* polarization experiment

*In vivo* AM polarization was carried out by administering cytokines into the nostrils of 12-week-old mice, under the Home Office License number P44492AC9. LPS (Sigma-Aldrich, 297–473–0) was used to induce M1 polarization (0.15 μg in 30 μL PBS). Interleukin-4c (IL-4c) was utilized to induce M2 polarization in alveolar macrophages. The IL-4c was prepared by combining recombinant IL-4 (PeproTech, 214-14) with an anti-IL-4 antibody (BD Biosciences, 554387) in a 1:5 ratio of IL-4 to antibody. Specifically, 0.05 μg of IL-4c was diluted in 30 μL of phosphate-buffered saline (PBS) for administration. Mice were given two doses of either PBS, IL-4c or LPS on days 0 and 2 under isoflurane-induced anesthesia. Mice were then culled by rising CO_2_ on day 4.

#### Primary cell isolation

Primary lung fibroblasts were isolated from mice aged between 2 and 3 months. Tissue was digested in 1000 U/mL bacterial collagenase type 4 (Gibco, 17104019) in trypsin (2.5 g/L), as previously described.^41^

AMs were isolated from 3-month-old mice by bronchoalveolar lavage (BAL), as previously described.^42^

#### Cell culture and *in vitro* cell differentiation

All monocytes/macrophages (primary or established cell lines) were maintained in culture in Roswell Park Memorial Institute (RPMI 1640, Sigma-Aldrich, R8758) culture medium containing 2 mM L-glutamine and 1.5 g/L sodium bicarbonate. The media was supplemented with 10% heat inactivated fetal bovine serum (FBS), 1% penicillin and streptomycin antibiotics (Sigma-Aldrich, P4333), 10 mM HEPES (Gibco, 15630-080) and 1 mM sodium pyruvate (Gibco, 11360-039).

All fibroblasts were maintained in Dulbecco’s Modified Eagle’s Medium F-12 (DMEM F-12, Gibco, 11320033) culture medium containing 2 mM L-glutamine. The media was supplemented with 10% heat inactivated FBS and 1% antibiotics. Cells were maintained at 37°C in a 5% CO_2_ humidified incubator.

Eph4 cells were maintained in Dulbecco’s Modified Eagle’s Medium (DMEM) with high glucose culture medium (Sigma-Aldrich, D5796), containing 4500 mg/L glucose, L-glutamine, and sodium bicarbonate, supplemented with 10% heat inactivated FBS and 1% antibiotics.

Unless otherwise stated, during experimentation all cells were cultured in the presence of 200 μM L-ascorbic acid. For co-culture/treatment experiments, fibroblasts were seeded onto either 8-well chamber slides or glass coverslips and allowed to adhere overnight. The media is then changed to to L-ascorbic acid containing media in the presence or absence of treatment (i.e., +/− macrophages, human TGFβ1 at 5 ng/mL from Peprotech 100-21, Avapritinib at 100 nM from Cambridge Bioscience HY-101561, KL001 from SLS SML1032). Cells were then cultured for 72 h before downstream processing, as described below.

*In vitro* THP-1 monocytes were differentiated into macrophages by 48 h incubation with 100 nM phorbol 12-myristate 13-acetate (PMA, Sigma Aldrich, P8139). Macrophages were polarized to M1 macrophages by incubation with 20 ng/mL of IFN-γ (Miltenyi Biotec, 130–096–481) and 10 ng/mL of LPS (Sigma-Aldrich, 297–473–0) for 24 h. Macrophages were polarized to M2 phenotype by incubation with 20 ng/mL of IL-4 (Miltenyi Biotec, 130–093–920) and 20 ng/mL of IL-13 (Miltenyi Biotec, 130–112–409) for 24 h.

### Method details

#### Preparation and collection of conditioned media (CM)

Cells were cultured for at least 5 days in serum-free media. CM was taken each day and passed through a 0.45 μM filter to remove cells and cell debris. The CM was then frozen until use.

#### Generation of PER2-Luc2 THP-1 cell line

PER2-Luc2 lentivirus was produced using HEK293T cells and transduced into THP-1 cells. Briefly, the PER2-Luc2 encoding lentivirus was produced by adding 1 mL Opti-MEM (Thermo Fisher Scientific, 31985062) to an Eppendorf with 2.5 μg psPAX2 packaging vector (Addgene, 12260), 2.5 μg pMD2.G envelope (Addgene,12259) and 5 μg per2-bre-luc2-ef1a-GFP transfer plasmid, which has been previously generated.^43^ This mixture was incubated at room temperature for 15 min. HEK293T cells were transfected with the lentivirus and incubated for at least 48 h before the virus was harvested. The viral supernatant was centrifuged at 500 × g, 4°C, for 5 min to pellet any remaining cells and then passed through a 0.45 μm polyethersulfone membrane filter. Virus transduction into THP-1 cells was achieved by incubating cells in a 6-well plate with 1 mL of the virus suspension and 8 μg/μL polybrene (Sigma Aldrich, TR-1003). The cells were incubated with the virus suspension for 48 h. The plasmid also contained a GFP tag, so fluorescence was observed using fluorescent microscopes to ensure that transduction of the plasmid was successful.

#### Generation of endogenously-tagged HiBiT-collagen lung fibroblasts

HiBiT-Col1a2 CRISPR-edited lung fibroblasts were genetically modified to endogenously express luminescently-tagged Col1a2 with a HiBiT tag at the end of the N-propeptide.^44^ This strategy for editing these cells has been shown to preserve collagen function while serving as an effective quantitative reporter for collagen.^45^^,^^46^

#### Generation of PDGFA knockout (KO) in THP-1 cell line

Cells were treated with CRISPR-Cas9 to delete the PDGFA gene. Two sgRNAs targeting PDGFA were designed, with sequences: cgggcacatgcttagtggca (g1) and catggaccccgtgagctctc (g2). The sgRNAs were introduced into the genome of THP-1 cells using CRISPR-Cas9. sgRNA and tracrRNA (IDT, 1072532) were combined in a 1:1 ratio before heating at 95°C for 5 min. The mixture was cooled to room temperature before centrifuging at 10,000 × g for 30 s. Cas9 (IDT, 1081058) was then added to the mixture at a 1:2 ratio and mixed. This was left for 10 min at room temperature to allow for annealing. The RNP complex was then delivered into THP-1 cells via electroporation using Neon NxT electroporation system (Thermo Fisher Scientific).

#### Circadian bioluminescence recording

The circadian rhythm of cells isolated from the *Per2*-Luc mouse and macrophages containing the PER2-Luc2 plasmid were measured using LumiCycle apparatus (Actimetrics), which provided real-time quantitative bioluminescence recordings. Traces were typically recorded for a minimum of 4 days. The LumiCycle analysis software (Actimetrics) performed baseline subtraction of recorded bioluminescence values using a 24-h moving average. Where applicable, 100 nM dexamethasone (dex) (Scientific Laboratory Supplies, D1756) was added to synchronize the cells’ circadian rhythm as a positive control. It was added to cell cultures in the LumiCycle following 24 h of recording (unless otherwise shown in figure) to establish how the circadian rhythm of a synchronized population should look like. Amplitude was calculated based on the second peak after treatment, using BioDare2 circadian analysis software.

Bioluminescence imaging before and after scratch wounding was conducted using an Obscura Alligator Luminescence System (Cairn Research), where image acquisition was achieved using MetaMorph (Molecular Devices) using the following settings: Digitizer 16-bit (1 MHz, EM Gain), EM Gain enabled at 1200, Vertical Shift Speed 230.95 KHz (4.33usec/pixel), Vertical Clock Voltage Normal, Pre-Amplifier Gain 2, Camera Shutter Open for Exposer, Exposure time 20 min, Binning 2, Camera Sate non-overlapped, Baseline offset 0. Confluent layer of lung fibroblasts were synchronized for an hour with 100 nM dexamethasone, before switching to recording media with 1% FBS/luciferin and put in the Alligator system. After 20 h, scratch wounds were introduced using a P200 tip. An image was taken every 25 min, and the resulting image series were analyzed using FIJI to measure bioluminescence signals.

#### HiBiT::Col1a2 luminescence readings

Primary murine lung fibroblasts expressing HiBiT::Col1a2 were cultured in 96-well plates (10,000 cells per well) for 48 h before addition of macrophage CM containing a total of 25 μg of protein. Fibroblasts were incubated with the CM for 4 days. CM was then removed, and cells were washed extensively with PBS before adding fresh media to determine collagen secretion. HiBiT::Col1a2 luminescence readings commenced after 48 h. Nano-Glo® HiBiT Extracellular Detection Kit (Promega, N2420) and a microplate reader (SYNERGY *neo*2, BioTek) were used to measure HiBiT-tagged Col1a2 in both the media from LF culture wells and the wells themselves, thus capturing secreted and deposited Col1a2, respectively.

#### Mass spectrometry proteomics analysis of CM

25 μg of protein per sample was prepared for Mass Spectrometry (MS)-based proteomics analysis using S-Trap columns (Protifi, C02-micro-10) where sequencing grade modified trypsin (Promega, V5111) was used for digestion following a previously published protocol.^47^ Porous Oligo R3 beads (Thermo Fisher Scientific, 1133907) were used to desalt peptides. Peptides were then dried in a vacuum centrifuge and resuspended in 10 μL of a 5% acetonitrile, 0.1% formic acid solution for MS analysis. MS data were analyzed using MaxQuant version 2.4.10.0 for label free quantification (LFQ) using data-dependent acquisition. Relative protein abundances were calculated. Mass spectrometry outputs (as LFQ intensity values) were further analyzed using Perseus version 2.0.11. Data were initially filtered to remove contaminants and decoy sequences, log2 transformed, and then samples grouped into experimental groups. Data were further filtered to remove entries in which none of the groups had at least two numeric values. Remaining “NaN” values generated by the log2 transformation were replaced by imputation from theoretical normal distribution, with a width of 0.3 and a down shift of 1.8. Inter-group differences were assessed using one-way ANOVA followed by post-hoc Turkey’s HSD test. Results with False Discovery Rate (FDR) less than 0.05 were considered significant. Further analysis and figure creation were carried out using Manchester Proteome Profiler (Web-based analysis platform developed at University of Manchester https://doi.org/10.64898/2026.05.14.725092). R packages were used to process protein intensity data and cluster analysis was performed to allow data visualization and identification of significantly enriched proteins. The DEP2 R package, which performs contaminant filtering, normalization and imputation of missing values was used. Normalization was performed using variance stabilizing transformation (VSN), with missing values imputed using the Missing Not At Random method (MNAR). A *p*-value of 0.05 and a fold change of 2 were chosen and applied to determine a list of 35 most significantly regulated proteins in each pairwise comparison.

#### RNA extraction, cDNA synthesis and quantitative PCR

To lyse cells for RNA extraction, cells were washed with PBS and snap frozen in liquid nitrogen with 250 μL TRIzol (Thermo Fisher Scientific, 15596026). RNA was isolated according to manufacturer’s instructions and RNA concentration was measured using NanoDrop One (Thermo Fisher Scientific). cDNA synthesis was performed using the TaqMan Reverse Transcription Kit (Thermo Fisher Scientific, N8080234). 500 ng–1000 ng of RNA was used per sample for cDNA synthesis, where the same RNA amount was used in all samples within the same experiment.

The qPCR master mix was made using 2× SYBR Green PCR Master Mix (Thermo Fisher Scientific, 4309155), 10 μM primer mix (forward and reverse) and nuclease-free water before addition of cDNA. Primer sequences are listed in Table S1.

#### Protein extraction and western blotting

CM was generated as described above, and protein concentration determined by Bradford assay. 25 μg of protein was used for western blot analysis. Briefly, samples were separated using SDS-PAGE using 4–12% Bolt Bis-Tris gel (Thermo Fisher Scientific). This is followed by transfer to nitrocellulose membranes using Biorad TransBlot® Turbo, blocked with 5% BSA in PBS for an hour, before incubation with PDGFA primary antibody (RayBiotech, 130–10142–20) at 1:500. This was followed by incubation with secondary antibody (Invitrogen, A21058) at a concentration of 1:10,000. The bound antibody signals were then detected using Licor CFX system. Ponceau red stain was used to confirm equal loading of samples.

#### Immunofluorescence, staining, microscopy and analysis

Cells plated on coverslips or 8-well μ-slides (Ibidi) were fixed with either 100% methanol at −20°C, or 4% PFA at 4°C, and then permeabilized with 0.2% Triton-X in PBS. Primary antibodies used were as follows: rabbit pAb collagen (1:400, Kerafast ENH018-FP), mouse mAb FN1 (1:400, Sigma F6140), mouse HiBiT pAb (Promega; N7200). Secondary antibodies conjugated to Alexa Fluor 488, Alexa Fluor 647, Cy3, and Cy5 were used (Thermo Scientific), and nuclei were counterstained with DAPI (Sigma). Coverslips were mounted using Fluoromount G (Southern Biotech).

Cells were imaged using an SP8 inverted confocal microscope (Leica) with an HC PL Apo 40×/1.30 oil objective. Settings were as follows: pinhole, 1 Airy unit; scan speed, 400 Hz bidirectional; format, 1,024 × 1,024. Sequential collection of images was used to eliminate crosstalk between channels. Quantification of collagen fibrils was done using ImageJ/FIJI software and achieved using a previously explained method (ISSN:1940–5901/IJCEM0059973). Briefly, collagen fibrils were quantified by splitting the acquired image into its separate channels and adjusting the threshold value so that only collagen fibrils were present. Once set, the threshold value remained consistent in all images. The area of collagen fibrils was then measured and normalized to nuclei number. Nuclei were counted using CellProfiler software.

#### Transcription binding site prediction

The transcription factor binding site prediction tool Contra v3^24^ (http://bioit2.irc.ugent.be/contra/v3) was used to search for binding sites in the promoter of PDGFA (chr5:139470907, number of introns: 5, NM_008808). The default (pragmatic) positional weight matrices (PWMs) score matching stringency in a 500 base promoter region was used. Search included E-box: Ebox, CLOCK/BMAL, Myc, Myc/Max. Cry1/Cry2 effectors are E-boxes/CLOCK/BMAL and ROR/REV-ERB. D-box: E4BP, NFIL3, ATF2, CEBP. GRE: GRE, NR3C1. ROR: RORA, RORB, RORC.

### Quantification and statistical analysis

Data are presented as specified in the figure legends. The sample size (N) represents the number of independent biological samples used in each experiment, whereas (n) represents the number of technical repeats. Details regarding sample size, experimental repeats, and statistical analyses are provided in the figure legends, or the Methods section. Data analysis was performed as described in the figure legends and was not conducted under blinded conditions in instances where software thresholding was performed for analysis.

Statistical significance was defined as *p* < 0.05. The significance levels are denoted as follows: ∗*p* < 0.05; ∗∗*p* < 0.01; ∗∗∗*p* < 0.001; ∗∗∗∗*p* < 0.0001; and n.s. (not significant). Actual *p*-values are reported where applicable. All statistical analyses were conducted using GraphPad Prism 10 software, or using alternative scripts (e.g., R scripts for proteomics analyses) as described in details in the STAR methods above.

## References

[1] Kadler K.E., Baldock C., Bella J., Boot-Handford R.P. (2007). Collagens at a glance. J. Cell Sci..

[2] Plikus M.V., Wang X., Sinha S., Forte E., Thompson S.M., Herzog E.L., Driskell R.R., Rosenthal N., Biernaskie J., Horsley V. (2021). Fibroblasts: Origins, definitions, and functions in health and disease. Cell.

[3] Wynn T.A., Chawla A., Pollard J.W. (2013). Macrophage biology in development, homeostasis and disease. Nature.

[4] Simões F.C., Cahill T.J., Kenyon A., Gavriouchkina D., Vieira J.M., Sun X., Pezzolla D., Ravaud C., Masmanian E., Weinberger M. (2020). Macrophages directly contribute collagen to scar formation during zebrafish heart regeneration and mouse heart repair. Nat. Commun..

[5] Chang J., Garva R., Pickard A., Yeung C.Y.C., Mallikarjun V., Swift J., Holmes D.F., Calverley B., Lu Y., Adamson A. (2020). Circadian control of the secretory pathway maintains collagen homeostasis. Nat. Cell Biol..

[6] Zhang R., Lahens N.F., Ballance H.I., Hughes M.E., Hogenesch J.B. (2014). A circadian gene expression atlas in mammals: implications for biology and medicine. Proc. Natl. Acad. Sci. USA.

[7] Mure L.S., Le H.D., Benegiamo G., Chang M.W., Rios L., Jillani N., Ngotho M., Kariuki T., Dkhissi-Benyahya O., Cooper H.M., Panda S. (2018). Diurnal transcriptome atlas of a primate across major neural and peripheral tissues. Science.

[8] Hoyle N.P., Seinkmane E., Putker M., Feeney K.A., Krogager T.P., Chesham J.E., Bray L.K., Thomas J.M., Dunn K., Blaikley J., O’Neill J.S. (2017). Circadian actin dynamics drive rhythmic fibroblast mobilization during wound healing. Sci. Transl. Med..

[9] Cunningham P.S., Meijer P., Nazgiewicz A., Anderson S.G., Borthwick L.A., Bagnall J., Kitchen G.B., Lodyga M., Begley N., Venkateswaran R.V. (2020). The circadian clock protein REVERBalpha inhibits pulmonary fibrosis development. Proc. Natl. Acad. Sci. USA.

[10] Wynn T.A., Vannella K.M. (2016). Macrophages in Tissue Repair, Regeneration, and Fibrosis. Immunity.

[11] Witherel C.E., Abebayehu D., Barker T.H., Spiller K.L. (2019). Macrophage and Fibroblast Interactions in Biomaterial-Mediated Fibrosis. Adv. Healthc. Mater..

[12] Viola A., Munari F., Sánchez-Rodríguez R., Scolaro T., Castegna A. (2019). The Metabolic Signature of Macrophage Responses. Front. Immunol..

[13] Mantovani A., Biswas S.K., Galdiero M.R., Sica A., Locati M. (2013). Macrophage plasticity and polarization in tissue repair and remodelling. J. Pathol..

[14] Yoo S.H., Yamazaki S., Lowrey P.L., Shimomura K., Ko C.H., Buhr E.D., Siepka S.M., Hong H.K., Oh W.J., Yoo O.J. (2004). PERIOD2::LUCIFERASE real-time reporting of circadian dynamics reveals persistent circadian oscillations in mouse peripheral tissues. Proc. Natl. Acad. Sci. USA.

[15] Martin-Burgos B., Wang W., William I., Tir S., Mohammad I., Javed R., Smith S., Cui Y., Arzavala J., Mora D. (2022). Methods for Detecting PER2:LUCIFERASE Bioluminescence Rhythms in Freely Moving Mice. J. Biol. Rhythms.

[16] Yamazaki S., Takahashi J.S. (2005). Real-time luminescence reporting of circadian gene expression in mammals. Methods Enzymol..

[17] Beta R.A.A., Arsenopoulou Z.V., Kanoura A., Dalkidis D., Avraamidou R., Balatsos N.A.A. (2022). Core clock regulators in dexamethasone-treated HEK 293T cells at 4 h intervals. BMC Res. Notes.

[18] Bhattacharya J., Westphalen K. (2016). Macrophage-epithelial interactions in pulmonary alveoli. Semin. Immunopathol..

[19] Sinha M., Lowell C. (2016). Isolation of Highly Pure Primary Mouse Alveolar Epithelial Type II Cells by Flow Cytometric Cell Sorting. Bio. Protoc..

[20] Hirota T., Lee J.W., St John P.C., Sawa M., Iwaisako K., Noguchi T., Pongsawakul P.Y., Sonntag T., Welsh D.K., Brenner D.A. (2012). Identification of small molecule activators of cryptochrome. Science.

[21] Hand L.E., Dickson S.H., Freemont A.J., Ray D.W., Gibbs J.E. (2019). The circadian regulator Bmal1 in joint mesenchymal cells regulates both joint development and inflammatory arthritis. Arthritis Res. Ther..

[22] Wang N., Liang H., Zen K. (2014). Molecular mechanisms that influence the macrophage m1-m2 polarization balance. Front. Immunol..

[23] Kazlauskas A. (2017). PDGFs and their receptors. Gene.

[24] Kreft Ł., Soete A., Hulpiau P., Botzki A., Saeys Y., De Bleser P. (2017). ConTra v3: a tool to identify transcription factor binding sites across species, update 2017. Nucleic Acids Res..

[25] Bowen-Pope D.F., Malpass T.W., Foster D.M., Ross R. (1984). Platelet-derived growth factor in vivo: levels, activity, and rate of clearance. Blood.

[26] Wu C.P., Lusvarghi S., Wang J.C., Hsiao S.H., Huang Y.H., Hung T.H., Ambudkar S.V. (2019). Avapritinib: A Selective Inhibitor of KIT and PDGFRalpha that Reverses ABCB1 and ABCG2-Mediated Multidrug Resistance in Cancer Cell Lines. Mol. Pharm..

[27] Burgoyne R.D., Morgan A. (2003). Secretory granule exocytosis. Physiol. Rev..

[28] Gope M.L., Gope R. (2009). Tyrosine phosphorylation of EGF-R and PDGF-R proteins during acute cutaneous wound healing process in mice. Wound Repair Regen..

[29] Chang J., Pickard A., Herrera J.A., O'Keefe S., Garva R., Hartshorn M., Hoyle A., Dingle L., Knox J., Jowitt T.A. (2025). Endocytic recycling is central to circadian collagen fibrillogenesis and disrupted in fibrosis. eLife.

[30] Shure D., Senior R.M., Griffin G.L., Deuel T.F. (1992). PDGF AA homodimers are potent chemoattractants for fibroblasts and neutrophils, and for monocytes activated by lymphocytes or cytokines. Biochem. Biophys. Res. Commun..

[31] Horikawa S., Ishii Y., Hamashima T., Yamamoto S., Mori H., Fujimori T., Shen J., Inoue R., Nishizono H., Itoh H. (2015). PDGFRalpha plays a crucial role in connective tissue remodeling. Sci. Rep..

[32] Andrae J., Gallini R., Betsholtz C. (2008). Role of platelet-derived growth factors in physiology and medicine. Genes Dev..

[33] Huang H., Mehta A., Kalmanovich J., Anand A., Bejarano M.C., Garg T., Khan N., Tonpouwo G.K., Shkodina A.D., Bardhan M. (2023). Immunological and inflammatory effects of infectious diseases in circadian rhythm disruption and future therapeutic directions. Mol. Biol. Rep..

[34] Dong Y., Lam S.M., Li Y., Li M.D., Shui G. (2025). The circadian clock at the intersection of metabolism and aging - emerging roles of metabolites. J. Genet. Genomics.

[35] O'Neill J.S., Hastings M.H. (2007). Circadian clocks: timely interference by microRNAs. Curr. Biol..

[36] Khalil W., Xia H., Bodempudi V., Kahm J., Hergert P., Smith K., Peterson M., Parker M., Herrera J., Bitterman P.B., Henke C.A. (2015). Pathologic Regulation of Collagen I by an Aberrant Protein Phosphatase 2A/Histone Deacetylase C4/MicroRNA-29 Signal Axis in Idiopathic Pulmonary Fibrosis Fibroblasts. Am. J. Respir. Cell Mol. Biol..

[37] Guillard J., Schwörer S. (2024). Metabolic control of collagen synthesis. Matrix Biol..

[38] Sancar A., Van Gelder R.N. (2021). Clocks, cancer, and chronochemotherapy. Science.

[39] Mukherji A., Tharaux P.L., Ray D.W., Baumert T.F. (2025). Perturbation of the circadian clock in chronic diseases involving organ fibrosis. J. Clin. Investig..

[40] Vitaterna M.H., King D.P., Chang A.M., Kornhauser J.M., Lowrey P.L., McDonald J.D., Dove W.F., Pinto L.H., Turek F.W., Takahashi J.S. (1994). Mutagenesis and mapping of a mouse gene, Clock, essential for circadian behavior. Science.

[41] Yeung C.Y.C., Zeef L.A.H., Lallyett C., Lu Y., Canty-Laird E.G., Kadler K.E. (2015). Chick tendon fibroblast transcriptome and shape depend on whether the cell has made its own collagen matrix. Sci. Rep..

[42] Van Hoecke L., Job E.R., Saelens X., Roose K. (2017). Bronchoalveolar Lavage of Murine Lungs to Analyze Inflammatory Cell Infiltration. J. Vis. Exp..

[43] Naven M.A., Zeef L.A.H., Li S., Humphreys P.A., Smith C.A., Pathiranage D., Cain S., Woods S., Bates N., Au M. (2022). Development of human cartilage circadian rhythm in a stem cell-chondrogenesis model. Theranostics.

[44] Calverley B.C., Kadler K.E., Pickard A. (2020). Dynamic High-Sensitivity Quantitation of Procollagen-I by Endogenous CRISPR-Cas9 NanoLuciferase Tagging. Cells.

[45] Schwinn M.K., Machleidt T., Zimmerman K., Eggers C.T., Dixon A.S., Hurst R., Hall M.P., Encell L.P., Binkowski B.F., Wood K.V. (2018). CRISPR-Mediated Tagging of Endogenous Proteins with a Luminescent Peptide. ACS Chem. Biol..

[46] Stevenson N.L., Bergen D.J.M., Lu Y., Prada-Sanchez M.E., Kadler K.E., Hammond C.L., Stephens D.J. (2021). Giantin is required for intracellular N-terminal processing of type I procollagen. J. Cell Biol..

[47] Herrera J.A., Mallikarjun V., Rosini S., Montero M.A., Lawless C., Warwood S., O’Cualain R., Knight D., Schwartz M.A., Swift J. (2020). Laser capture microdissection coupled mass spectrometry (LCM-MS) for spatially resolved analysis of formalin-fixed and stained human lung tissues. Clin. Proteomics.

