# Supplementary material for: Macrophages promote collagen deposition through circadian regulation of fibroblasts
Source: iScience. 2026 Sep 21;29(10):117573. doi: 10.1016/j.isci.2026.117573 (PMC13626760; doi:10.1016/j.isci.2026.117573)
Supplement: Supplementary file 1 [file mmc1.pdf]

iScience, Volume *29*

Supplemental information

Macrophages promote collagen deposition through circadian regulation of fibroblasts

Katherine Lowles, Xin-Yang Zhang, Xue-Feng Yao, Marie F.A. Cutiongco, Joshua J. Hughes, Shi-Yang Li, John Knox, Madeleine Coy, Wei-Hsiang Lin, Andrew S. MacDonald, Karl E. Kadler, Qing-Jun Meng, Tracy Hussell, and Joan Chang

Supplementary Figure 1

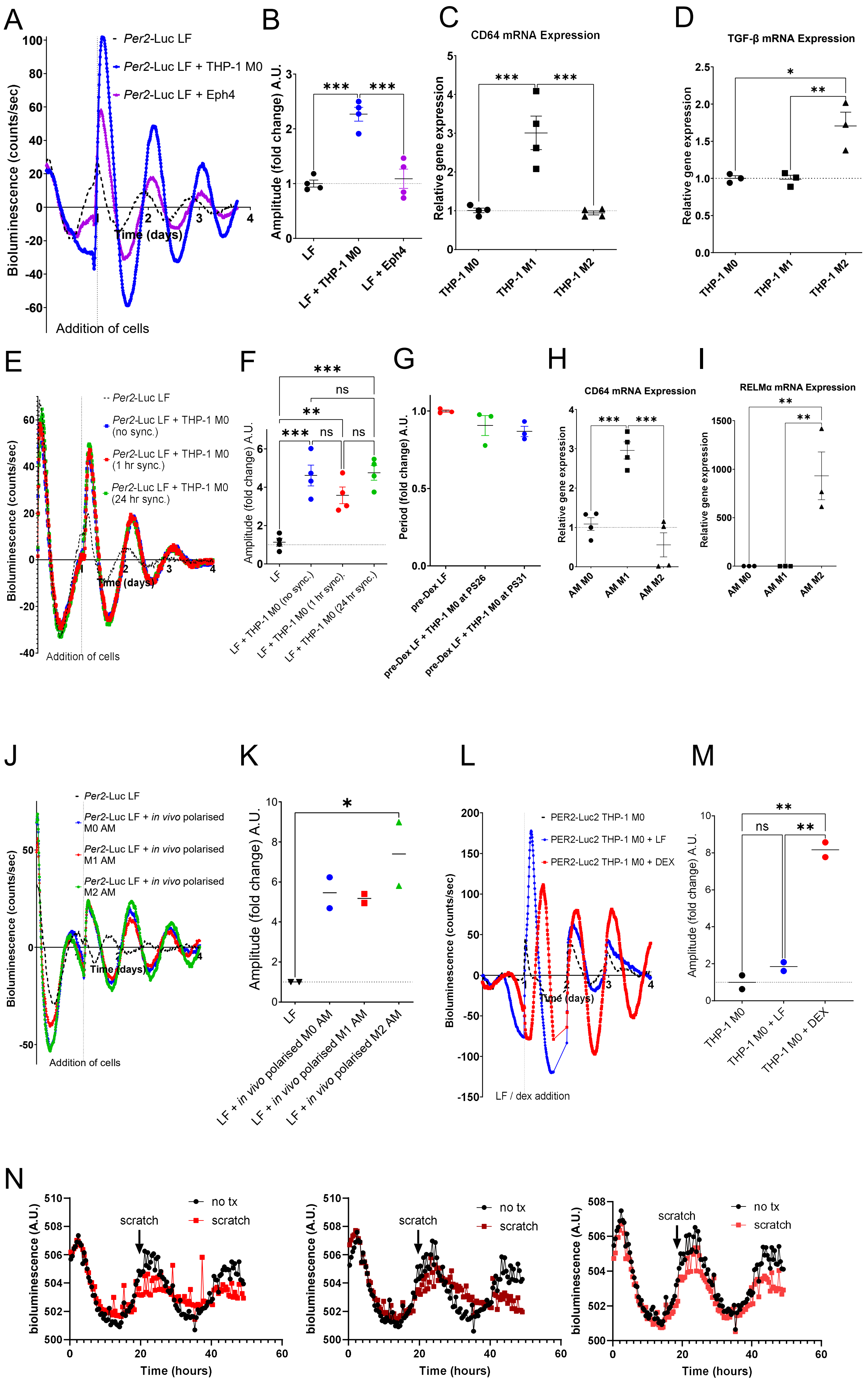

Supplementary Figure 1: Bioluminescence traces and validation of macrophage polarisation states by qPCR

(A) Addition of THP-1 macrophages induced a robust circadian rhythm in fibroblasts, while Eph4 cells did not. Representative of N = 4.

(B) Amplitude fold change was shown to be significant following addition of THP-1 macrophages, compared to LF only, whereas Eph4 did not markedly increase circadian amplitude in fibroblasts. Data are represented as mean +/- SEM. N = 4 biological repeats, ***p<0.001, one-way ANOVA.

(C) qPCR analysis of M1 gene markers in polarised human cell line THP 1 macrophages. Compared to M0 and M2-polarisation states, M1-polarised THP-1 macrophages expressed significantly higher CD64. Data are represented as mean +/- SEM. N = 4 biological repeats, ***p<0.001, one-way ANOVA.

(D) qPCR analysis of M2 gene marker in polarised human cell line THP 1 macrophages. Compared to M0 and M1-polarisation states, M2-polarised macrophages expressed significantly higher TGF-β. Data are represented as mean +/- SEM. N = 3 biological repeats, *p<0.05, **p<0.01, one-way ANOVA.

(E) Bioluminescence recordings of *Per2*-Luc lung fibroblasts showed macrophage synchronisation status does not affect the degree to which circadian amplitude is increased in fibroblasts. Non-synchronised, 1 hour synchronised, and 24-hour synchronised macrophages all induced a similar increase in circadian amplitude in fibroblasts compared to non-synchronised macrophages. Representative of N = 4.

(F) All macrophage synchronisation states significantly increased circadian amplitude in fibroblasts, but there was no statistical difference when comparing synchronisation states to each other. Data are represented as mean +/- SEM. N = 4 biological repeats, **p<0.01, ***p<0.001, one-way ANOVA.

(G) Period analysis revealed no change in the length of period between LF monoculture, and those with THP-1 M0 added at different phase of the circadian rhythms. Data are represented as mean +/- SEM, one-way ANOVA.

(H) qPCR analysis of M1 gene markers in polarised murine primary AMs. Compared to M0 and M2-polarisation states, M1-polarised THP-1 macrophages expressed significantly higher CD64 compared to M0 and M2. Data are represented as mean +/- SEM. N = 4 biological repeats, ***p<0.001, one-way ANOVA.

(I) qPCR analysis of M2 gene markers in polarised murine primary AMs. Compared to M0 and M2-polarisation states, M2 polarised macrophages expressed significantly higher RELMα. Data are represented as mean +/- SEM. N = 3 biological repeats, **p<0.01.

(J) Addition of *in vivo* polarised AMs induced a robust circadian rhythm in fibroblasts, while Eph4 cells did not. Representative of N = 2.

(K) Amplitude fold change was shown to be significant following addition of M2-polarised AM, compared to LF only. Data are represented as median. N = 2 biological repeats, *p<0.01, one-way ANOVA.

(L) Fibroblasts were added to PER2-Luc2 THP-1 macrophages but failed to induce a robust rhythm. Representative of N = 2.

(M) Amplitude differences when fibroblasts and dexamethasone were added to macrophages was quantified, showing fibroblasts do not significantly increase macrophage circadian rhythm amplitude, but dexamethasone does. Data are represented as median. N = 2 biological repeats; **p< 0.01, one-way ANOVA.

(N) Bioluminescence imaging and quantification over time revealed that synchronised fibroblasts (no treatment, no tx) maintain a strong circadian rhythm over 48 hours. When a scratch wound is introduced (scratch) at 20 hours there is a dampening of the Per2luc signal. Shown here are the 3 individual biological repeats.

Supplementary Figure 2

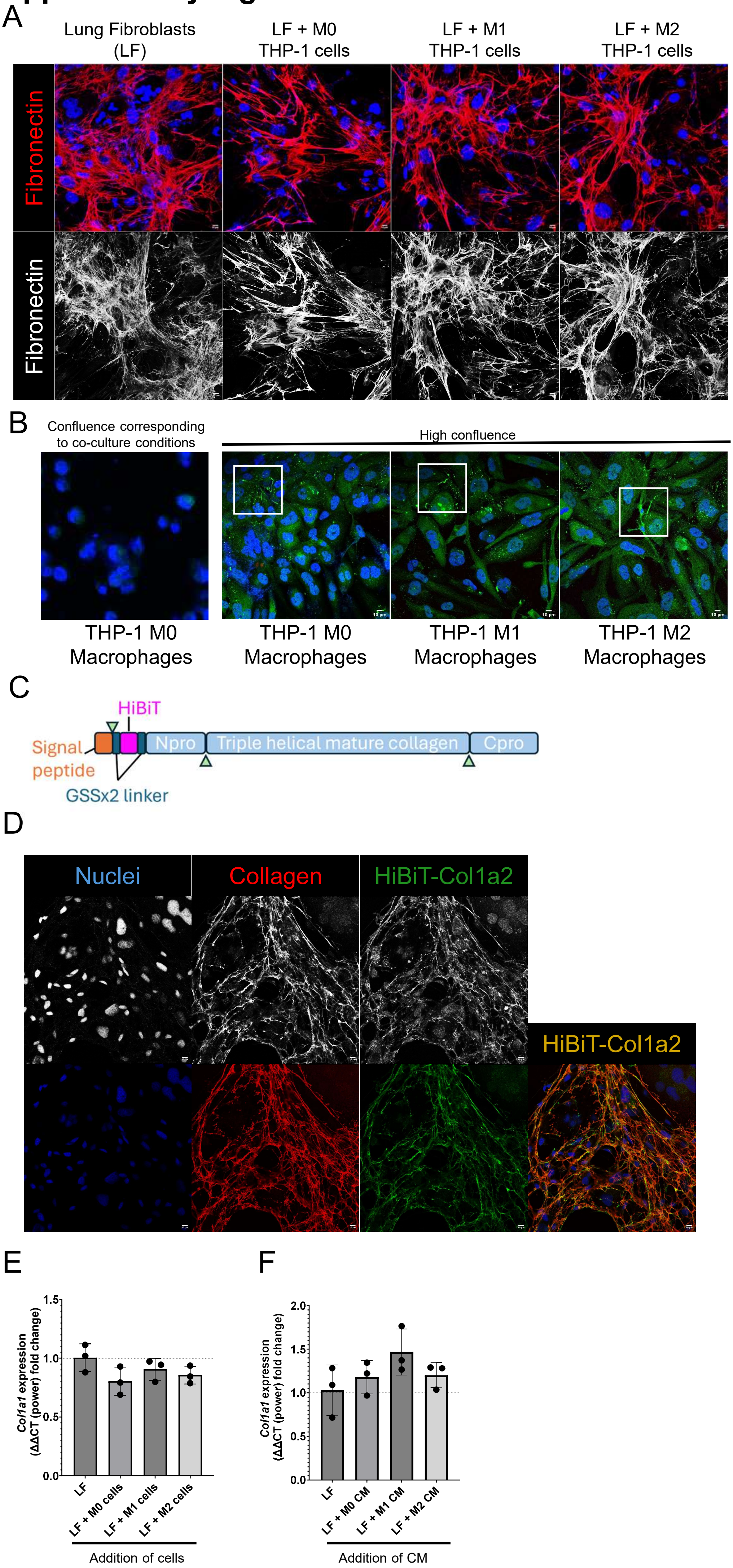

Supplementary Figure 2. Fibronectin staining in co-cultures, collagen deposition by macrophages and co-staining of collagen and HibiT-Col1a2.

(A) Fibronectin staining in co-cultures of fibroblasts with polarised THP-1 macrophages. No macrophage-induced changes in fibronectin deposition by fibroblasts were observed. Nuclei were stained with DAPI. Scale bar = 10 μm. Representative of N = 3.

(B) IF images of M0, M1 and M2 THP-1 macrophages depositing collagen fibrils (green). THP-1 cells were cultured for 6 days before fixing and staining. Collagen fibrils are surrounded by a white box. Nuclei were stained with DAPI. Scale bar = 10 μm. Representative of N = 3 biological repeats.

(C) Schematic of the HiBT-*Col1a2* tag at the Npro region of the *Col1a2* gene.

(D) Co-staining of collagen (red) and HiBiT-Col1a2 (green) in HiBiT-Col1a2 lung fibroblast cultures. Nuclei were stained with DAPI. Merge image shows overlap of collagen HiBiT-Col1a2 and collagen in yellow. Scale bar = 10 μm. Representative of N = 3 biological repeats.

(E) qPCR analysis of *Col1a1* expression levels in LF cultured with macrophage cells showed no significant change. Data are represented as mean +/- SD. N = 3 biological repeats, one-way ANOVA.

(F) qPCR analysis of *Col1a1* expression levels in LF cultured with macrophage-derived CM showed no significant change. Data are represented as mean +/- SD. N = 3 biological repeats, one-way ANOVA.

Supplementary Figure 3

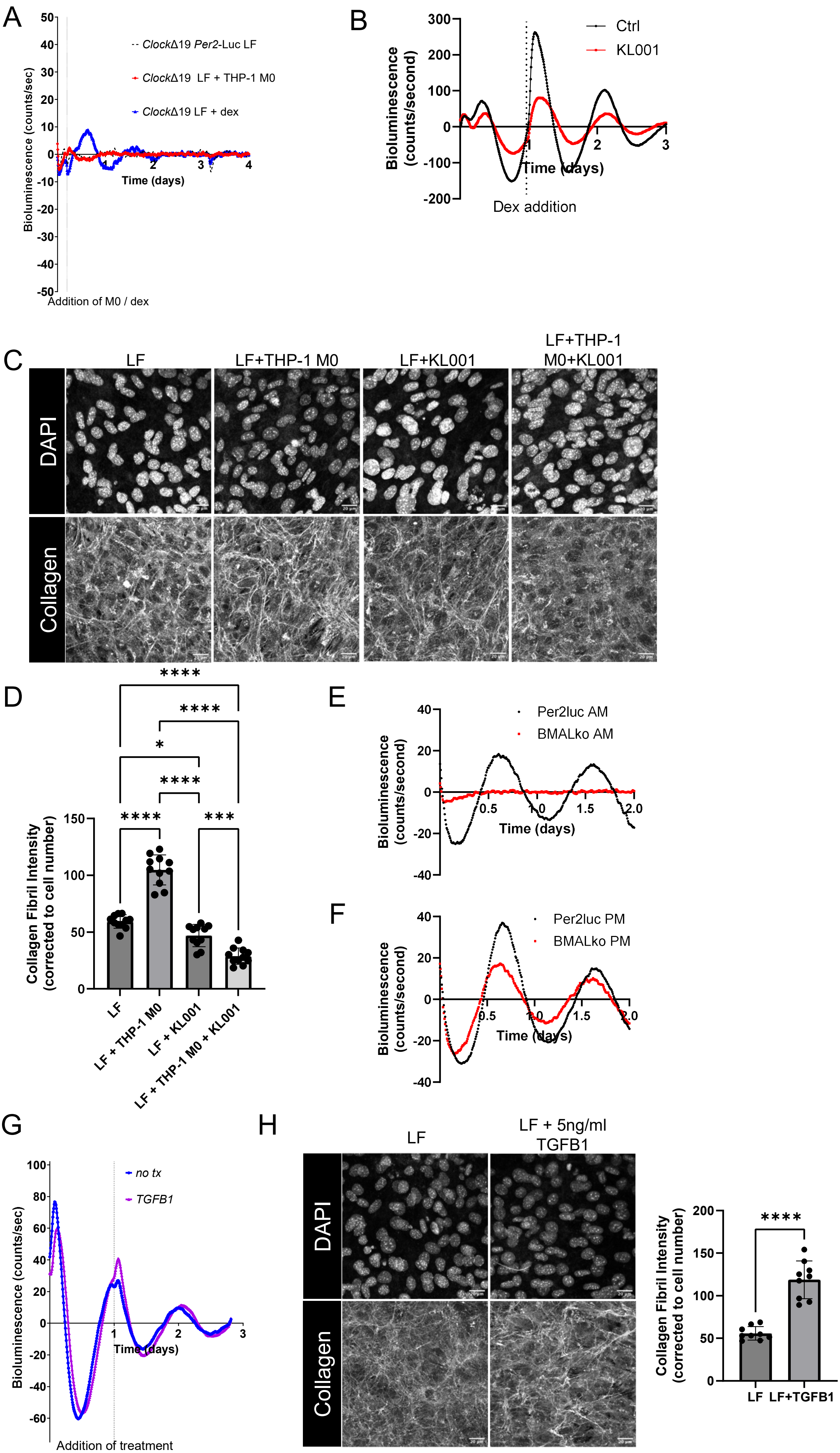

Supplementary Figure 3. Bioluminescence traces of *Clock*Δ19 *Per2*-Luc LF.

(A) THP-1 M0 and dexamethasone treatments did not induce a circadian rhythm in fibroblasts with an impaired clock. Representative of N = 3 biological repeats.

(B) Addition of 5μM KL001 led to a reduction of circadian rhythms. Representative of N = 4 biological repeats.

(C) IF images of lung fibroblasts in monoculture and co-culture with THP-1 M0 macrophages, in presence or absence of 5μM KL001. Top panel shows DAPI for nuclei, and bottom panel shows collagen staining. Representative of N = 3 biological repeats. Scale bar = 20 μm.

(D) Quantification of collagen fibril intensity revealed that KL001 treatment abrogated M0 macrophage promoting effects on collagen deposition. Data are represented as mean +/- SD. n = 12 technical repeats across N = 3 biological repeats. $^{*}p<0.05$, $^{***}p<0.001$, $^{****}p<0.0001$, one-way ANOVA.

(E) Circadian traces of Per2luc primary alveolar macrophages (AM) and Col6a1-Cre BMALko AM, showing that the circadian rhythm is knocked out in these AMs. Representative of N = 2 mice.

(F) Circadian traces of Per2luc primary peritoneal macrophages (PM) and Col6a1-Cre BMALko PM, showing that the circadian rhythm is dampened in these PMs. Representative of N = 2 mice.

(G) Lumicycle recording of fibroblasts with 5 ng/mL TGFβ1 added at 24 hours after recording. No discernible changes was observed between treated and untreated (no tx) cells. Representative of N = 3 biological repeats.

(H) Left: IF images of fibroblasts with or without TGFβ1 treatment. Top panel shows DAPI staining for nuclei, bottom panel shows collagen-I staining. Right: quantification of collagen fibril intensity revealed that TGFβ1 treatment increased collagen deposition. Data are represented as mean +/- SD. n = 9 across N = 3 biological repeats, unpaired two-tail t-test.

Supplementary Figure 4

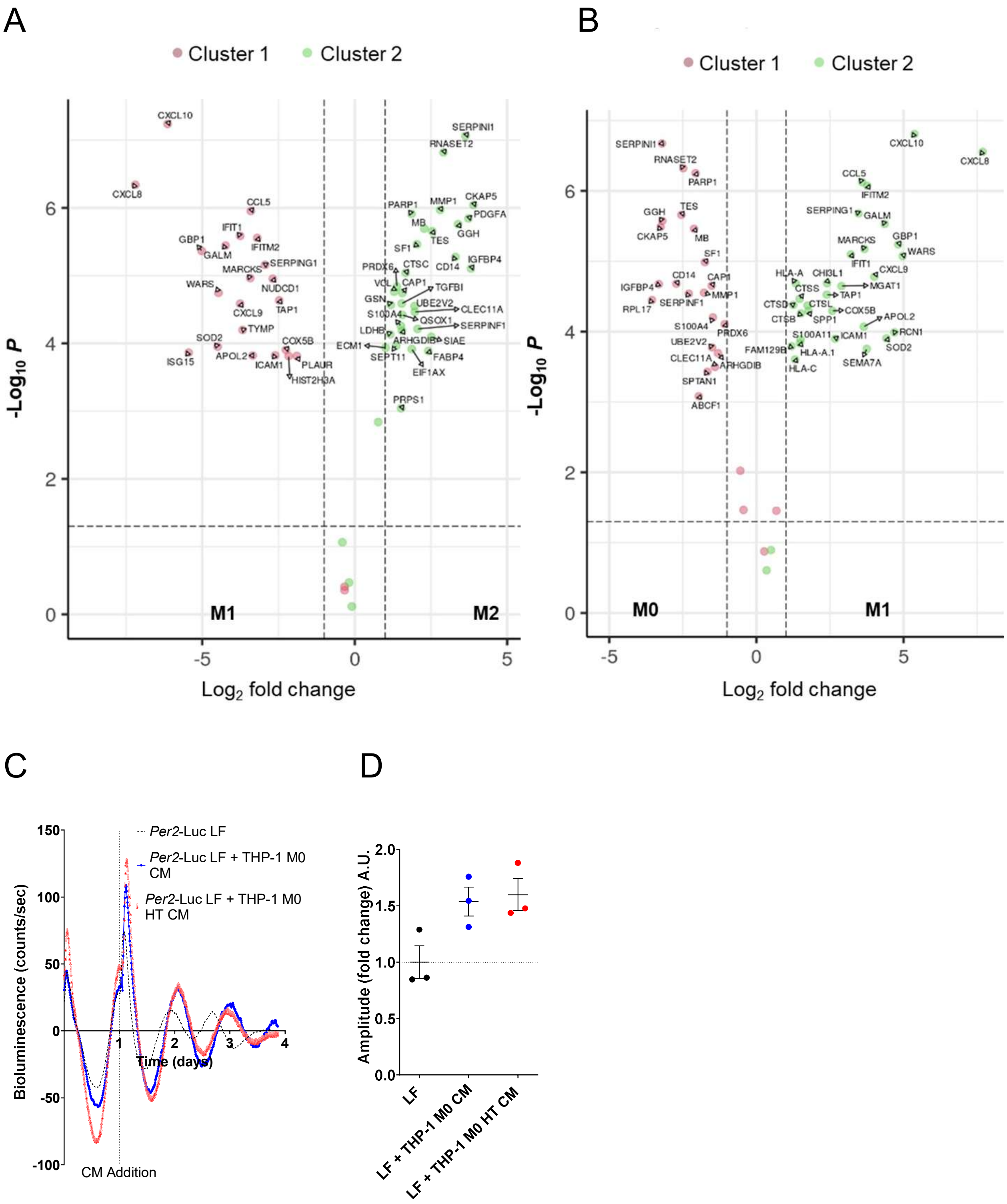

Supplementary Figure 4: Pairwise comparisons of proteins present in polarised macrophage CM.

(A) Volcano plots comparing proteins present in M2 CM vs M1 CM. Parameters of volcano plots: fold change cutoff = ≥ 2, and p-value ≤ 0.05. N = 3 biological repeats.

(B) Volcano plots comparing proteins present in M1 CM vs M0 CM. Parameters of volcano plots: fold change cutoff = ≥ 2, and p-value ≤ 0.05. N = 3 biological repeats.

(C) Bioluminescence recordings of *Per2*-Luc lung fibroblasts with the addition of HT CM showed that HT THP-1 macrophage CM induced a circadian rhythm comparable to that observed with untreated CM. Representative of N= 3.

(D) The difference in circadian amplitude changes between the addition of HT CM and untreated CM was found to be statistically insignificant. Data are represented as mean +/- SEM. N=3 biological repeats, one-way ANOVA.

Supplementary Figure 5

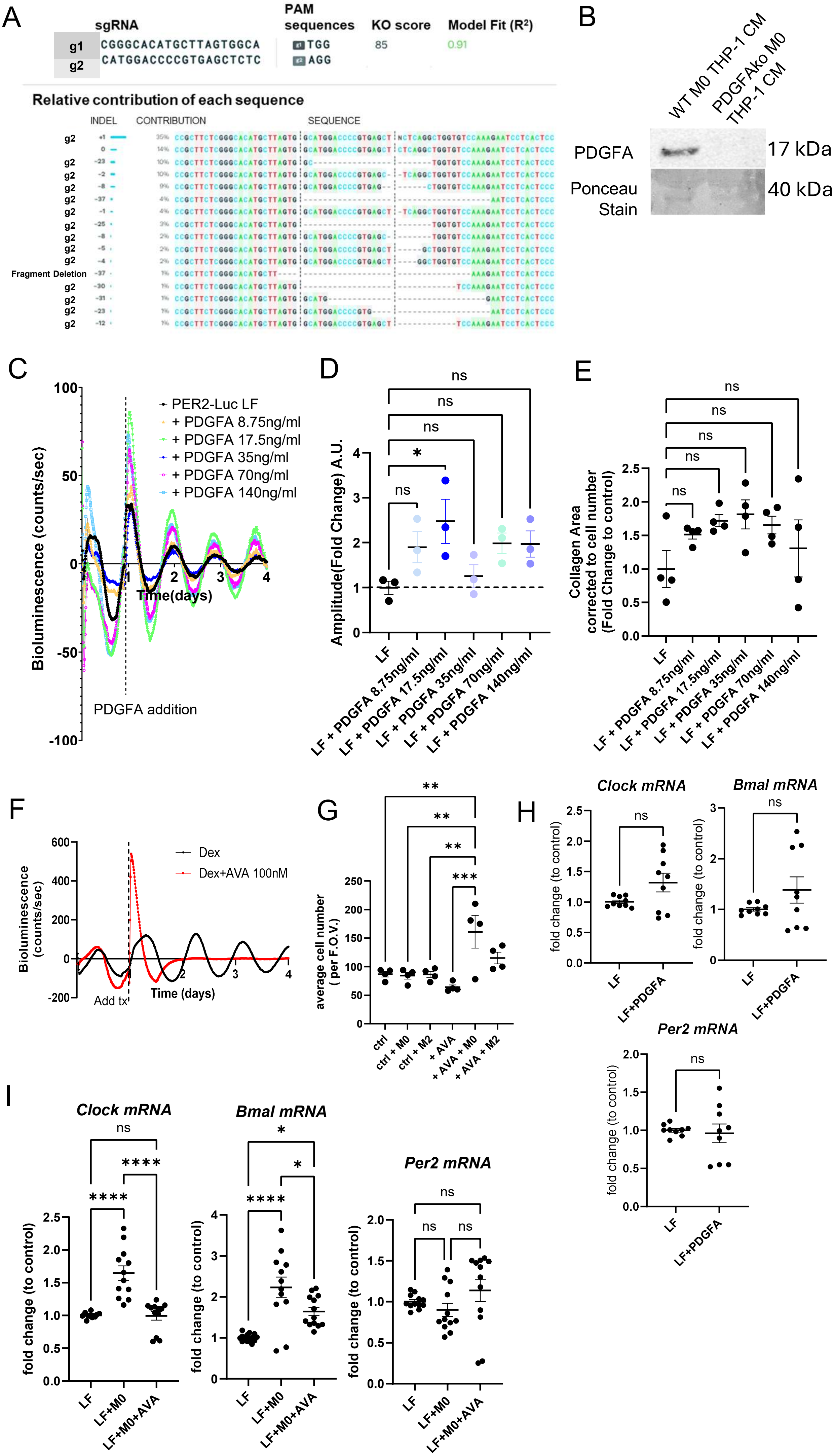

Supplementary Figure 5: CRISPR-Cas9 gene editing analysis of PDGFA knockout in THP-1 cell line.

(A) CRISPR-Cas9 gene editing analysis of PDGFA knockout in THP-1 cell line An 85% PDGFA knockout was achieved in THP-1 cells. The resultant cell population was mixed, with different InDels contributing to the knockout.

(B) Western blot staining of PDGFA in WT M0 THP-1 and PDGFAko M0 THP-1 CM. Ponceau stain used as a loading control. Representative of N=3.

(C) Bioluminescence recordings of *Per2*-Luc lung fibroblasts with the addition of different doses of PDGFA. Representative of N= 3.

(D) The difference in circadian amplitude changes between the different doses of PDGFA was found to be statistically insignificant apart from 17.5ng/ml dose and no dose-dependent response was observed. Data are represented as mean +/- SEM. N=3 biological repeats, one-way ANOVA.

(E) Quantification of immunofluorescence images on collagen deposition showed that recombinant PDGFA did not significantly increase collagen deposition in fibroblasts at all doses. Data are represented as mean +/- SEM. n = 4 technical repeats, one-way ANOVA.

(F) Bioluminescence recordings of pre-synchronised *Per2*-Luc lung fibroblasts with the addition of 100 nM Avapritinib (AVA) abrogated the circadian rhythm compared to that control cells (0.1% DMSO treatment). Representative of N= 4.

(G) Number of cells in fibroblast mono- or co-culture with M0/M2 THP-1 macrophages, with or without AVA treatment. Controls (ctrl) were treated with 0.1% DMSO vehicle. Most conditions have similar number of cells apart from fibroblasts co-cultured with M0 THP-1 and AVA, where there is significantly higher number of cells. Data are represented as mean +/- SEM. N = 4 biological repeats, **$p<0.01$, ***$p<0.001$, Welch's t-test.

(H) *Clock, Bmal1, Per2* expression levels in lung fibroblasts (LF) compared to PDGFA-treated cultures (LF+PDGFA). None of the three core clock genes showed significantly different expressions. Data are represented as mean +/- SEM. n = 9 technical repeats across N = 3 biological repeats, Welch's t-test.

(I) *Clock, Bmal1, Per2* expression levels in lung fibroblasts (LF) compared to LF co-cultured with M0 THP-1 macrophages in presence of 0.1% DMSO (LF + M0) and co-culture in presence of avapritinib (LF + M0 + AVA). In the case for *Bmal* and

Clock, presence of macrophages increased expression levels, which is then inhibited in presence of AVA. Data are represented as mean +/- SEM. n = 12 technical repeats across N = 4 biological repeats. One-way ANOVA.

Table S1. List of oligonucleotides use in this paper

Oligonucleotide type	Designation	Source or reference	Sequence
guide RNA	Human PDGFA knockout	IDT	G1: cgggcacatgcttagtggca<br>G2: catggaccccgtgagctctc
qPCR primer	Mouse *18s*	Sigma-Aldrich	F: GTAACCCGTTGAACCCCATT<br>R: CCATCCAATCGGTAGTAGCG
qPCR primer	Mouse *Cd64*	Sigma-Aldrich	F: GTCGGTGGGGAAGTGGTTAAAT<br>R: CCCCTCACACCATAAAGTGAC
qPCR primer	Mouse *Col1a1*	Sigma-Aldrich	F: GCCTGCTTCGTGTAAACTCC<br>R: TTGGTTTTTGGTCACGTTCA
qPCR primer	Mouse *Relm-a*	Sigma-Aldrich	F: CAAGGAACTTCTTGCCAATCCAG<br>R: CCAAGATCCACAGGCAAAGCCA
qPCR primer	Mouse *Clock*	Sigma-Aldrich	F: GGCTGAAAGACGACGAGAAC<br>R: GGTGTTGAGGAAGGGTCTGA
qPCR primer	Mouse *Bmal*	Sigma-Aldrich	F: GGACTATTACCAGGGCAGGC<br>R: ATGTGGGGGTTCTCACCAAG
qPCR primer	Mouse *Per2*	Sigma-Aldrich	F: AGAGTGTGGTGTCCCTCACC<br>R: ATGTGCAGCCAGAACTTCCT
qPCR primer	Human *18S*	Sigma-Aldrich	F: GAGACTCTGGCATGCTAACTAG<br>R: GGACATCTAAGGGCATCACAG
qPCR primer	Human *CD64*	Sigma-Aldrich	F: AACTCTGCTCCTTTGGGTTCC<br>R: CTCTTGGAACACGCTGACCC
qPCR primer	Human *TGFB1*	Sigma-Aldrich	F: CACGTGGAGCTGTACCAGAA<br>R: GAACCCGTTGATGTCCACTT
